# Impact of Response Shift on Time to Deterioration in Quality of Life Scores in Breast Cancer Patients

**DOI:** 10.1371/journal.pone.0096848

**Published:** 2014-05-14

**Authors:** Zeinab Hamidou, Tienhan S. Dabakuyo-Yonli, Francis Guillemin, Thierry Conroy, Michel Velten, Damien Jolly, Sylvain Causeret, Olivier Graesslin, Mélanie Gauthier, Mariette Mercier, Franck. Bonnetain

**Affiliations:** 1 Quality of life and Cancer clinical research Platform, Marseille, France; 2 Public health laboratory, EA 3279-College of Medicine, Marseille, France; 3 Biostatistic and Quality of Life Unit, Centre Georges François Leclerc, Dijon, France; 4 EA 4184, College of Medicine, Dijon, France; 5 Inserm, CIC-EC, department of clinical epidemiology and evaluation, university hospital of Nancy, Nancy, France; 6 Medical oncology department, Centre Alexis Vautrin, Nancy, France; 7 Epidemiology and public health laboratory, College of Medicine, Strasbourg, France; 8 University hospital of Reims, Reims, France; 9 Surgery department, Centre Georges François Leclerc, Dijon, France; 10 Gynecology and Obstetrics Department, Mother Child Institute, University hospital of Reims, Reims, France; 11 Cellular and Molecular Biology Laboratory, University hospital of Besançon, Besançon, France; 12 Methodology and quality of Life in Oncology unit (EA 3181), University hospital of Besançon, Besançon, France; University of Louisville, United States of America

## Abstract

**Background:**

This prospective multicenter study aimed to study the impact of the recalibration component of response-shift (RS) on time to deterioration (TTD) in health related quality of life (QoL) scores in breast cancer (BC) patients and the influence of baseline QoL expectations on TTD.

**Methods:**

The EORTC-QLQ-C30 and BR-23 questionnaires were used to assess the QoL in a prospective multicenter study at inclusion (T0), at the end of the first hospitalization (T1) and, three (T2) and 6 months after the first hospitalization (T3). Recalibration was investigated by the then-test method. QoL expectancy was assessed at diagnosis. Deterioration was defined as a 5-point decrease in QoL scores, considered a minimal clinically important difference (MCID). TTD was estimated using the Kaplan-Meier method. Cox regression analyses were used to identify factors influencing TTD.

**Results:**

From February 2006 to February 2008, 381 women were included. Recalibration of breast cancer patients' internal standards in the assessment of their QoL had an impact on TTD. Median TTD were significantly shorter when recalibration was not taken into account than when recalibration was taken into account for global health, role-functioning, social-functioning, body-image and side effects of systemic therapy. Cox multivariate analyses showed that for body image, when recalibration was taken into account, radiotherapy was associated with a shorter TTD (HR: 0.60[0.38–0.94], whereas, no significant impact of surgery type on TTD was observed. For global health, cognitive and social functioning dimensions, patients expecting a deterioration in their QoL at baseline had a significantly shorter TTD.

**Conclusions:**

Our results showed that RS and baseline QoL expectations were associated with time to deterioration in breast cancer patients.

## Introduction

The assessment of longitudinal changes in subjective patient-reported outcomes such as health-related quality of life (HRQoL) is a key component of many clinical and research evaluations. Indeed, the aim of assessing the impact of disease and treatment on HRQoL is increasingly stressed as crucial for evaluating the overall treatment effectiveness in cancer clinical trials. Moreover, cancer patients require information not only related to survival estimates, but also regarding HRQoL issues [1].

The challenge of using HRQoL measurements in longitudinal studies or clinical trials is related to their self-report nature and also to their subjectivity. Because measurements of HRQoL are completed from the patient's perspective, they could be modified by psychological phenomena such as health expectancies [2], [3]. For instance, the mechanism by which people assess or quantify their HRQoL could change over time. These changes, which are closely related to the process of accommodating to the illness, are referred to as response shift (RS) [4]–[6]. Schwartz and Sprangers defined response shift through three components “as a change in the meaning of one's self-evaluation of a target construct as a result of a change in the respondent's internal standards of measurement (recalibration), a change in the respondent's values (reprioritization) or a redefinition of the target construct (reconceptualization)[5]. A major goal of measuring patient-reported HRQoL is to determine to what extent changes in HRQoL scores over time represent true changes in HRQoL due to treatment or cancer and to what extent they reflect measurement error [7]. The occurrence of RS has been demonstrated in breast cancer (BC) patients [8]–[10]. Response shift is a natural process that could distort the interpretation of change in HRQoL scores over time in interventional comparative studies. Characterizing response shift may therefore be a requirement to obtain a valid and sensitive assessment of change over time.

Another concern in assessing HRQoL is how to deal with missing data [11] since they could impact the results of HRQoL estimates and lead to biased interpretations. Indeed, in longitudinal studies, observations of patients can be missed at certain time points because they miss visits or do not fill in some questionnaires. In these cases, the interpretation of HRQoL results can be seriously hampered by these missing data. Thus, analysis methods requiring complete cases (e.g., multivariate analysis of variance) are not adequate. Analysis methods should retain, at least, all of the available data [11] but should produce results that are robust and meaningful for clinicians in order to help decision making [12], [13]. In this way, the time to deterioration in QoL scores (TTD) approach has been defined as a method of longitudinal analysis for breast cancer (BC) patients [14]. Indeed, TTD can deal with missing data by making underlying assumptions about whether the missing data reflect a deterioration of the patient's health status or not. Furthermore, the measure of TTD might be more familiar to clinicians because it is based on Kaplan–Meier survival curves and hazard ratios (HR)[15].

The aims of this study were to evaluate the impact of the recalibration component of RS on TTD estimations in patients with BC. The secondary objective was to examine the influence of baseline QoL expectations on TTD in patients with BC.

## Methods

### Patients

A prospective multicenter randomized cohort study that included all women hospitalized for the diagnosis or treatment of primary BC or for a suspicion of BC was implemented in the cancer centers of Dijon, and Nancy, and in the university hospitals of Strasbourg and Reims. Patients were included between February 2006 and February 2008. Patients with other primary cancer sites than BC and patients already hospitalized or treated for BC were excluded. Only, women patients were included. Patients who declined the study or who were unable to give a written informed consent were excluded.

All of the participants gave their written informed consent, and the protocol of the study was approved by the regional ethics committee (Comité Consultatif de Protection des Personnes dans la Recherche Biomédicale de Bourgogne) in 2005.

### Health related Quality of life assessments

HRQoL was assessed using the EORTC-QLQ-C30 [16] and the EORTC QLQ-BR23 questionnaires [17] at diagnosis (T0), at the end of the first hospitalization (T1) and, three (T2) and six months after the first hospitalization (T3). The QLQ-C30 is a cancer specific tool composed of 30 items which generate 15 scores: five scores of functional parameters, a financial difficulties scale, and eight scores for symptoms. The breast cancer module comprises 23 questions assessing disease symptoms and the side-effects of treatment. These scores vary from 0 (worst) to 100 (best) for functional functions and from 0 (best) to 100 (worst) for symptom parameters.

Patients were also asked to assess their QoL expectations at baseline using the following question: do you expect that your QoL:

1. will not change globally,
2. will deteriorate,
3. will improve.

### Assessment of the recalibration component of RS using the then-test method

**Figure 1:**
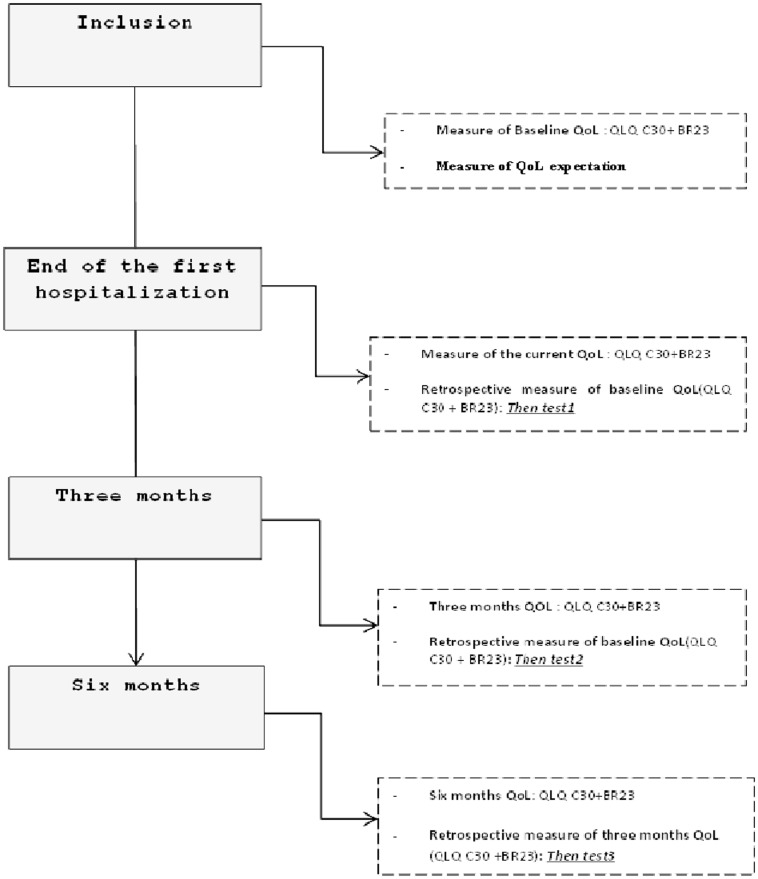
Quality of life data collection procedure.

Recalibration was assessed using the then-test method. This method requires patients to rate their previous health state from their current perspective [5], [18]. The order in which the QoL questionnaires, then-test and post-test, were administered was determined by randomization 1∶1 with center stratification to assess the impact of the order of the questionnaires on RS estimates. In arm A, patients were asked to complete the questionnaires at time T (posttest), and then retrospectively (then-test) to assess baseline QoL at the end of the first hospitalization. In arm B, the order of the questionnaires was then-test/post-test. In this study, we did not compare patients according to randomized arm because previous study showed only a small impact of the ordre of the randomized arm on QoL scores [10]. Three then-tests were implemented (figure 1): two to retrospectively assess baseline QoL (measured at the end of the first hospitalization and 3 months after the first hospitalization) and one to retrospectively assess the three-month QoL (measured at 6 months). In other words, patients were asked to retrospectively assess their baseline QoL at T1 (then test1) and at T2 (then test 2), and to retrospectively assess their three-month QoL at T3 (then test 3).The mean differences between the assessment of the baseline QoL at the inclusion (pretest) and then test1 were calculated to assess recalibration at the end of the first hospitalization. In order to assess recalibration at 3 months, the mean differences between the assessment of the baseline QoL at the inclusion and thentest2 were calculated. Lastly, the mean differences between the three-month QoL and its retrospective assessment at 6 months (then-test3) were compared in other to assess recalibration in internal standards at 6 months. A + (or −) mean difference between the “then-test and the pre-test” retrospectively reflects a higher (or lower) QoL level at baseline (or at 3 months) for the functional (or symptoms) dimensions.

### Statistical methods

Patients' characteristics were described and compared according to the completion of baseline questionnaire in order to determine whether missing score at inclusion was dependent on patients' clinical or sociodemographic status.

Wilcoxon matched pairs tests were used to assess recalibration.

#### Time to QoL deterioration

All patients who had a baseline and at least one follow-up QoL assessment were included in the TTD analyses.

The time to QoL deterioration (TTD) was defined as the time from inclusion in the study to the first 5-point [19] decrease in QoL scores according to baseline score. Patients were censored at the time of the last QoL completed if they had not deteriorated before that [14].

To take into account the recalibration component of RS, then-test assessments were used as reference scores when significant recalibration effects were observed. Therefore, if significant recalibration of baseline QoL was observed only at T1 (or at T2), analyses were done using then-test1 (or then-test2), as the reference score. In addition, then-test3 was used in TTD analyses (instead of three-month QoL), when significant recalibration of the three-month QoL was observed at T3.

The TTD was estimated using the Kaplan-Meier method. The TTD was described using medians and the 95% confidence interval (CI). Statistical significant difference between median TTD when recalibration component of RS was taken into account and median TTD when recalibration was not taken into account was assessed using bootstrap Kaplan-Meier estimate of median TTD. Nonparametric 95% confidence intervals for the difference in bootstrap Kaplan-Meier estimate of median TTD were computed. Differences between medians were considered statistically significant if their 95% confidence intervals did not include the value of 0.

Cox regressions were applied to identify factors associated with TTD for each QoL dimension. All variables with an univariate Cox p value ≤0.20 were eligible for multivariate Cox analyses. Cox multivariate analyses were stratified on the center of inclusion. The statistical significance level was set at p = 0.05 for Cox models analyses and reduced to p = 0.01 for the analysis performed with the then-test method in order to prevent false positive results due to the number of multiple comparisons performed with this method.

Analyses were performed using STATA Statistics 11/Data Analysis Software (StataCorp LP, College Station, Texas, USA)

## Results

### Patients

Between February 2006 and February 2008, 381 patients were included. Patients' characteristics have been widely described elsewhere [10]. Briefly, the mean age was 58 years (SD = 11.1), 124 (33%) patients underwent mastectomy, 131(34%) had sentinel lymph node biopsy (SLNB) and 155 (40%) received adjuvant chemotherapy (Table 1).

**Table 1:** Characteristics of patients according to the completion of quality of life questionnaire at baseline.

	Patients with at least one baseline score (359)		Patients without baseline score (22)		Fisher exact test
	N	(%)	N	(%)	
**Lymph node dissection(LND)**					
Axillary LND	127	35.4	11	50	0.363
Sentinel lymph node biopsy	124	34.5	7	31.8	
ALND+SLNB	32	8.9	0	0	
No LND	72	20.1	3	13.6	
unknown	4	1.1	4	4.6	
**Surgery type**					1.000
mastectmoy	117	32.6	7	31.8	
no mastectomy	227	63.2	14	63.6	
unknown	15	4.2	1	4.6	
**Chemotherapy**					0.500
yes	148	41.2	7	31.8	
no	204	56.8	14	63.6	
unknown	7	2	1	4.6	
**Radiotherapy**					0.814
yes	239	66.6	15	68.2	
no	113	31.5	6	27.3	
unknown	7	2	1	4.6	
**Hormone therapy**					0.509
yes	162	45.1	8	36.4	
no	190	52.9	13	59.1	
unknown	7	2	1	4.6	
**Care center**					0.030
Dijon	250	69.6	21	95.5	
Nancy	74	20.6	0	0	
Reims	17	4.7	1	4.6	
Strasbourg	18	5	0	0	
**Comorbidity**					0.162
yes	229	63.8	10	45.5	
no	128	35.7	11	50	
unknown	2	0.6	1	4.6	
**Stage (AJCC)**					0.555
0	76	21.2	2	9.1	
1	126	35.1	9	40.9	
2	111	30.9	7	31.8	
3_4	15	4.2	0	0	
unknown	31	8.6	4	18.2	
**Marital status**					0.309
married	262	73	19	86.4	
Not married	87	24.2	3	13.6	
unknown	10	2.8	0	0	
**Live alone**					0.391
yes	59	16.4	2	9.1	
no	260	72.4	19	86.4	
unknown	40	11.1	1	4.6	
**Education Degree**				0.204	
low level	156	43.5	11	50	
high level	150	41.8	5	22.7	
unknown	53	14.8	6	27.3	
**Job**					0.491
working	173	48.19	8	36.36	
not working	172	47.91	12	54.55	
unknown	14	3.9	2	9.1	
	**Mean**	**SD**	**Mean**	**SD**	**p Mann&Whitney**
**Age**	57.8	11.1	61.3	9.9	

### QoL completion

At baseline, 359 (94.2%) patients completed the questionnaire with at least one available QoL dimension and 357 (93.7%) had a baseline and at least one follow-up QoL assessment. The clinical and pathological characteristics of these two populations were similar and are presented in Table 1. Only the center of inclusion was statistically different according to missing score.

### Response shift

### Retrospective assessments of baseline QoL

The occurrence of recalibration effects differed according to the time of the retrospective assessment (T1 or T2) for 7 dimensions. For fatigue, appetite loss and the side effects of systemic therapy, with mean differences (MD) in QoL scores of −1.8(p = 0.0006), −2.9(p = 0.0081) and −1.96(p = 0.0001), respectively (Table 2), symptoms were significantly higher at inclusion than the retrospective assessment at T1 (then-test1). These differences were no longer statistically significant with the retrospective assessment of the baseline QoL at T2 (then-test 2).

**Table 2:** Significant changes in internal standards.

	QoL at baseline(Pre-test)		Then-Test 1-minus-Pre-test		Then-Test 2-minus-Pre-test		QoL at 3 months		Then-Test 3-minus-QoL at 3 months		
	N	Mean (SD)	Mean (SD)	p*	Mean (SD)	p*	N	Mean (SD)	N	Mean (SD)	p*
**QLQ-C30**											
Global Health	285	69.1(19.2)	−0.6 (16.9)	0.8397	−3.7(18.2)	**0.0014** †	294	62.3(20.7)	294	−0.3(21.5)	0.8990
Physical	291	91.0 (14.1)	−0.3(10.2)	0.1876	−1.6(12.6)	0.1826	301	82.3(16.5)	301	4.3(14.3)	**<0.0001**
Role	293	89.2(20.4)	−2.6(19.0)	0.3109	−6.3(22.2)	**<0.0001** †	297	72.4(28.7)	297	6.9(28.5)	**0.0003**
Emotional	289	64.0(25.7)	6.0(18.9)	**<0.0001**	6.8(21.1)	**<0.0001**	298	73.3(25.2)	298	−3.6(23.9)	**0.0067**
Cognitive	291	82.6(21.2)	3.1(15.3)	**0.0001**	3.0(18.2)	**0.0018**	297	79.7(22.87)	297	3.5(20.3)	**0.0016**
Social	276	90.1(18.9)	−0.3(17.0)	0.6823	−3.8(19.3)	**0.0012** †	294	77.3(26.5)	294	4.7(25.4)	**0.0063**
Fatigue	287	23.3(23.3)	−1.8(18.0)	**0.0006**	1.37(20.8)	0.4479†	298	37.6(26.0)	298	−9.4(25.3)	**<0.0001**
Nausea	292	3.9(12.6)	−0.9(8.3)	0.0763	1.3(15.7)	0.0763†	296	9.3(17.9)	296	−3.2(19.5)	**0.0085**
Pain	296	13.7(21.7)	0.9(19.0)	0.1668	2.5(22.1)	0.0641	302	24.2(25.5)	302	−5.0(23.5)	**0.0001**
Dyspnea	287	11.7(21.1)	−1.8(15.4)	0.0399	−0.4(16.2)	0.6978	297	15.9(23.5)	297	−3.25(23.5)	**0.0057**
Insomnia	285	37.6 (31.4)	−5.3(26.8)	**0.0003**	−7.2(30.8)	**0.0001**	294	36.3(31.3)	294	−3.9(33.0)	**0.0099**
Appetite Loss	286	11.7(23.1)	−2.9(18.8)	**0.0081**	−2.7(23.5)	0.0551	294	14.6(24.8)	294	−4.4(23.9)	**0.0029**
Diarrhea	286	8.5(17.2)	−3.3(11.8)	**<0.0001**	−2.9(16.8)	**0.0019**	291	7.7(17.2)	291	−0.4(21.6)	0.2057
**QLQ-BR23**											
Body image	266	90.2(17.6)	−0.3(12.2)	0.4919	−6.4(21.4)	**0.0001** †	295	71.8(30.9)	295	6.9(24.7)	**<0.0001**
Sexual functioning	228	26.0(24.7)	0.7(13.4)	0.5037	1.6(15.9)	0.1000	250	22.2(22.5)	250	4(19.2)	**0.0022**
Future perspectives	266	48.2(29.9)	8.0(29.8)	**<0.0001**	7.6(32.3)	**<0.0001**	295	56.8(32.3)	295	−1.1(32.2)	0.2573
Systemic therapy side effects	284	13.0(15.6)	−1.9(9.6)	**0.0001**	0.1(13.2)	0.7163†	297	23.3(19.5)	297	−7.5(19.1)	**<0.0001**
Breast symptoms	242	11.7(15.4)	−0.6(14.2)	0.1013	2.2(19.3)	0.3315	299	24.7(23.0)	299	−7.0(20.3)	**<0.0001**
Arm symptoms	272	7.7(13.9)	1.7(18.5)	0.5953	2.6 (16.2)	0.0521	299	15.7(18.3)	299	−2.3(17.2)	**0.0077**

Then-test 1: retrospective assessment of baseline at the end of 1st hospitalization.

Then-test 2: retrospective assessment of baseline at 3 months.

Then-test 3: retrospective assessment of 3-month QoL.

† significant difference between then-test 1 and then-test 2 with a Wilcoxon matched pairs tests p<0.01.

SD: standard deviation.

*Wilcoxon matched pairs tests p value.

Moreover, the MD in global health (GHS), role-functioning and social-functioning scores as well as body-image were not statistically significant with then-test1, but became significant with then-test2. Indeed, QoL scores for GHS, role-functioning, social-functioning and body-image were significantly higher at inclusion than the retrospective assessment at T2: MD = −3.7(p =  0.0014), MD = −6.3(p<0.0001), MD = −3.86(p = 0.0012) and MD = −6.47(p = 0.0001), respectively (Table 2).

For emotional-functioning, cognitive-functioning, future perspectives, diarrhea and insomnia symptoms, the recalibration effects at T1 and T2 were similar (Table 2). QoL scores were higher for the retrospective assessments as compared to the baseline QoL level. MD between then-test2 and pretest scores were MD = 6.89(p<0.0001), MD = 3.09(p = 0.0012) and MD = 7.6(p<0.0001) for emotional-functioning, cognitive-functioning and future perspectives, respectively. Insomnia: MD = −7.25(p = 0.0001) and diarrhea: MD = −2.9(p = 0.0019) symptoms were significantly higher at inclusion the retrospective assessment (Table 2).

#### Retrospective assessment of the three months QoL (then-test3)

The retrospective assessment of 3-month QoL scores at T3 (then-test3), showed that MD between then-test 3 and 3-month scores were statistically different for most QoL dimensions (Table 2). For example, QoL scores were higher at the retrospective assessments (T3) than at 3 months for physical-functioning (MD = 4.3) and role-functioning (MD = 6.95). Furthermore, fatigue (MD = −9.48), pain (MD = −5.07), dyspnea (MD = −3.25) and insomnia (MD = −3.96) symptoms were significantly higher at 3 months than when evaluated retrospectively at T3.

### Time to QoL deterioration

**Figure 2:**
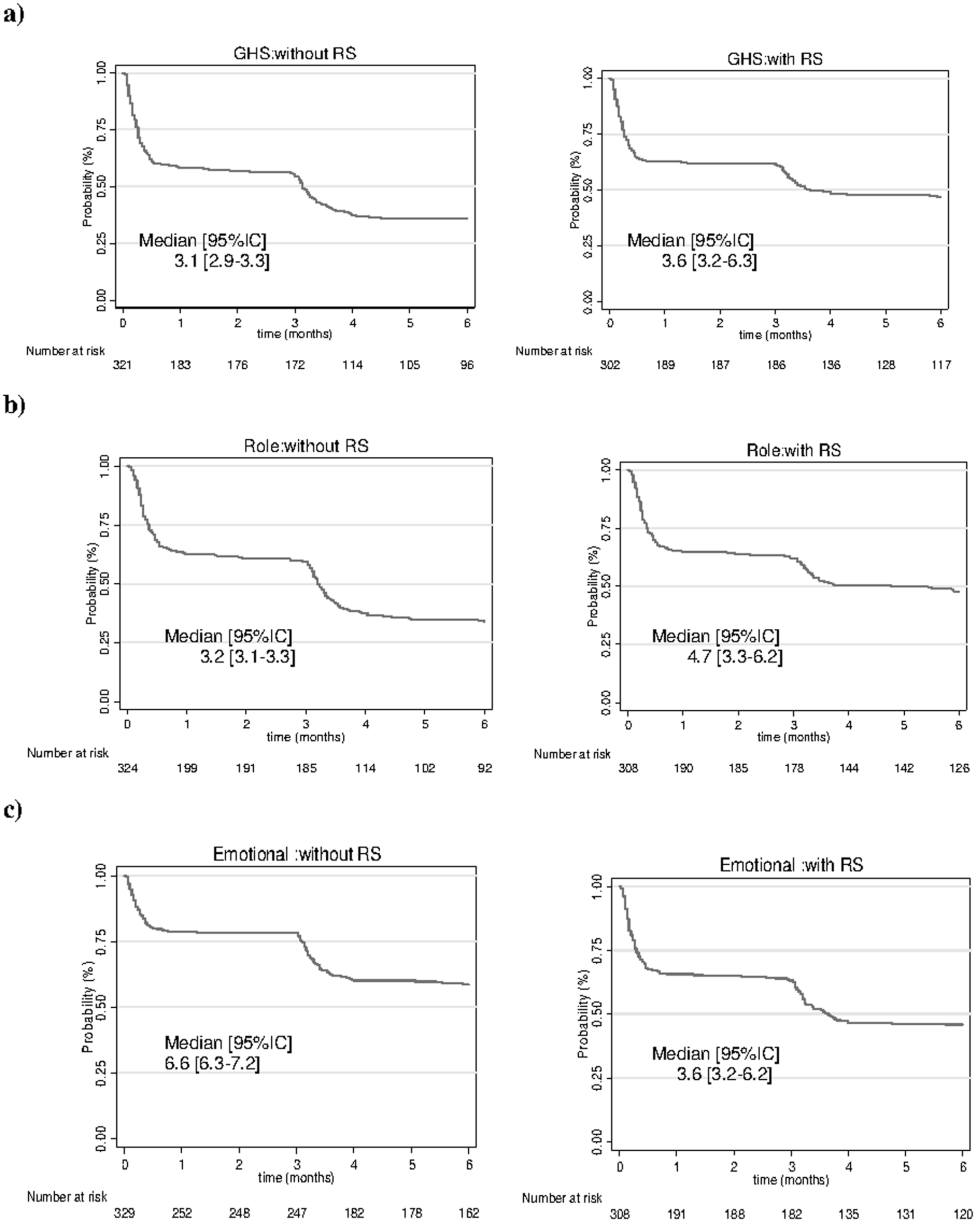
Time to deterioration in QoL score in the studied population with response shift (RS) taken into account or not. a) for the general health score of the QLQ-C30. b) for the role functioning score of the QLQ-C30. c) for emotional functioning. d)for social functioning. e) for body image. f) for systematic therapy side effects.

Medians TTD for the studied population are shown in table 3. Results showed that median TTD were significantly shorter when recalibration was not taken into account than when recalibration was taken into account for global health, role-functioning, social-functioning, body-image and side effects of systemic therapy (figure 2 a to f). For example for GHS, the median TTD increased from 3.1[2.9–3.3] when recalibration was not taken into account to 3.6[3.2–6.3] when it was (figure 2a). For role-functioning (figure 2b), the median TTD increased from 3.2[3.1–3.3] to 4.7[3.3–6.2] when recalibration was taken into account. For social functioning score (figure 2d), median TTD increased from 3.6 months to 6.3 months when recalibration was taken into account. For body image score (figure 2e), median TTD increased from 3.3 months to 6.2 months (table 3).

**Table 3:** Median TTD according recalibration of response shift.

	Without taking recalibration of RS into account				With recalibration component of RS taken into account				Difference in bootstrap Kaplan-Meier estimate of median TTD	
	n	event	median	CI	n	event	Median	CI	Difference	CI
QLQ-C30										
Global Health	321	216	3.1	[2.9–3.3]	302	171	3.6	[3.2–6.3]†	0.43	[0.1–3.1]
Physical	329	249	0.5	[0.4–2.2]	327	233	0,53	[0.4–1,4]‡	−0.03	[−1.9–0.5]
Role	324	226	3.2	[3.1–3.3]	308	191	4.7	[3.3–6.2]???	0.60	[0.03–2.9]
Emotional	329	153	6.6	[6.3–7.2]	308	173	3.6	[3.2–6.2]???	−3.13	[−3.8–−0.5]
Cognitive	328	154	7.1	[6.1–8.6]	307	139	6.6	[6.3–NR]???	−0.53	[−Inf Inf]
Social	325	190	3.6	[3.3–6.1]	305	154	6.3	[6.1–6.7]???	2.63	[0.2–3.2]
Fatigue	324	241	2.9	[0.6–3.1]	325	305	2,86	[0.5–3.1]¥	−0.16	[−2.4–2.3]
Nausea	331	121	8.2	[6.6–NR]	329	90	12.2	[12.2–NR]‡	NR	[NR–NR]
Pain	331	230	3.1	[0.9–3.3]	330	217	3.0	[0.7–3.2]‡	−0.06	[−2.3–1.4]
Dyspnea	327	123	7.2	[6.8–8.1]	307	106	6.9	[6.7–7.5]¥	−0.25	[−1.1–0.2]
Insomnia	326	138	7.2	[6.5–8.6]	301	141	6.6	[6.4–7.2]???	NR	[NR–NR]
Appetite Loss	330	106	8.6	[7.3–NR]	306	87	8.6	[7.3–NR]¥	NR	[NR–NR]
Constipation	327	142	7.8	[6.3–NR]	327	124	7.8	[6.9–NR]‡	0.21	[−1.6–NR]
Diarrhea	328	59	NR	[8.2–NR]	309	69	NR	[8.2–NR]†	NR	[NR–NR]
Financial	318	72	NR	[9.5–NR]	NA	NA	NA	NA	NA	NA
QLQ-BR23										
Body image	307	204	3.3	[3.2–3.5]	326	301	6,2	[6,0–6,5]???	3.0	[2.7–3.2]
Sexual functioning	284	95	8.6	[7.2–NR]	277	90	9.8	[6.9–NR]‡	2.8	[−Inf–Inf]
Sexual enjoyment	128	55	6.5	[6.0–NR]		NA	NA	NA	NA	NA
Future perspectives	311	88	7.8	[7.2–NR]	300	114	7.8	[7.2–NR]†	NR	[NR–NR]
Systemic therapy side effects	324	191	3.6	[3.4–4.3]	328	308	6,16	[4,2–6.4]¥	2.56	[0.4–2.9]
Breast symptoms	280	223	0.8	[0.5–3]	277	205	0.7	[0.4–3]‡	−0.05	[−2.2–1.2]
Arm symptoms	308	208	3	[0.7–3.3]	309	205	3	[0.7–3.5]‡	−0.01	[−2.4–2.4]
Upset by hair loss	44	17	6	[3.6–NR]	NA	NA	NA	NA	NA	NA

Then-test 1: retrospective assessment of baseline at the end of 1st hospitalization.

Then-test 2: retrospective assessment of baseline at 3 months.

Then-test 3: retrospective assessment of 3-month QoL.

† then-test2.

¥ then-test1 & then-test3 (i.e. significant recalibration was also observed at the retrospective assessment of 3-month QoL).

? then-test2 & then-test3 (i.e. significant recalibration was also observed at the retrospective assessment of 3-month QoL).

‡ then-test3.

NA: Not applicable (no significant recalibration).

NR: Not reached.

Inf: Infinite.

However, for emotional-functioning dimension (figure 2c) median TTD was significantly longer when recalibration was not taken into account. Bootstrap Kaplan-Meier estimate of difference in median TTD was −3.13[−3.8–−0.5]. For the other dimensions no statistically significant difference was found between median TTD.

### Univariate analyses of TTD

Results of the univariate Cox analyses of TTD are reported in table S1 in File S1 for QLQ-C-30 score and table S2 in File S1 for QLQ-BR23 scores. An MCID of 5 points was used for these analyses. For example, for the body-image score, when recalibration was not taken into account, there was no beneficial effect on TTD of either SLNB or not undergoing radiotherapy. When recalibration was taken into account, women treated with SLNB had a significantly longer TTD than those treated with axillary lymph node dissection (ALND): HR = 0.65[0.45–0.93]. Concerning radiotherapy, patients who did not receive treatment by radiotherapy had a significantly longer TTD than those who underwent radiotherapy.

### Cox multivariate analyses of TTD

Multivariate Cox models analyses were done for all dimensions of the QLQ-C30 and BR-23 questionnaire. However, for parsimony of the presentation, only dimensions (of QLQ-C30 or BR23) where times to deterioration estimations were significantly influenced by factors are shown in table 4. For body-image, when RS was not taken into account, cox multivariate analyses showed that the modality of surgery was significantly associated with TTD. Patients who underwent mastectomy had a shorter TTD for body-image as compared to patient having conservative surgery: HR 1.8[1.3–2.5].

**Table 4:** Multivariate cox regression analyses of time to QoL score deterioration for factors influencing significantly TTD.

	N (event)	Hazard ratio	(95%°CI)	p		N (event)	Hazard ratio	(95%°CI)	p
	Without taking recalibration of RS into account					With recalibration of RS taken into account			
					**Body image**				
**Surgery type**	**286(195)**					272(143)			
no mastectmoy		1					1		
mastectomy		1.83	[1.32–2.55]	*<0.001*			1.44	[0.97–2.14]	*0.065*
**Lymph node dissection(LND)**									
Axillary LND		1					1		
Sentinel lymph node biopsy		1.02	[0.72–1.44]	*0.892*			0.70	[0.46–1.05]	*0.088*
ALND+SLNB		0.83	0.45–1.53]	*0.565*			0.63	[0.29–1.35]	*0.242*
No LND		0.59	0.37–0.96]	*0.034*			0.75	[0.42–1.35]	*0.344*
**Radiotherapy**									
yes							1		
no							0.60	[0.38–0.94]	*0.028*
					**Arm symptoms**				
**Lymph node dissection(LND)**						292(195)			
Axillary LND		1					1		
Sentinel lymph node biopsy		0.59	[0.42–0.84]	*0.003*			0.64	[0.45–0.91]	*0.014*
ALND+SLNB		1.08	[0.63–1.85]	*0.76*			1.14	[0.66–1.99]	*0.624*
No LND		0.56	[0.35–0.92]	*0.019*			0.62	[0.37–1.04]	*0.073*
					**Nausea**				
**Lymph node dissection(LND)**	**324(119)**					303(87)			
Axillary LND		1					1		
Sentinel lymph node biopsy		0.4	[0.25–0.64]	*0.043*			0.48	[0.27–0.84]	*0.01*
ALND+SLNB		0.76	[0.39–1.47]	*0.442*			0.33	[0.11–0.98]	*0.048*
No LND		0.67	[0.39–1.14]	*0.854*			0.94	[0.49–1.79]	*0.928*
**Comorbidity**									
yes		1					1		
no		0.61	[0.40–0.91]	*0.019*			0.48	[0.29–0.79]	*0.004*
**Age(years)**									
<58		1					1		
> = 58		0.55	[0.37–0.83]	*0.012*			0.59	[0.36–0.94]	*0.028*
**social status**									
couple							1		
single							0.44	[0.24–0.82]	*0.01*
**Quality of life expectations**									
improvement		1					1		
deterioration		1.71	[1.09–2.2.68]	*0.017*			1.25	[0.730–2.14]	*0.409*
no change		0.95	[0.60–1.50]	*0.841*			0.9	[0.53–1.54]	*0.726*
					**cognitive functioning**				
**Quality of life expectations**						**301(137)**			
improvement							1		
deterioration							1.64	[1.07–2.52]	*0.021*
no change							1.02	[0.67–1.53]	*0.923*
					**breast symptoms**				
**Lymph node dissection(LND)**	**268(216)**					264(199			
Axillary LND		1					1		
Sentinel lymph node biopsy		1.46	[1.04–2.04]	*0.027*			1.25	[0.88–1.77]	*0.204*
ALND+SLNB		2.36	[1.37–4.07]	*0.002*			2.16	[1.23–3.76]	*0.007*
No LND		1.12	[0.73–1.71]	*0.598*			0.9	[0.57–1.42]	*0.667*
**Professional status**									
working		1					1		
non-working		0.68	[0.49–0.93]	*0.019*			0.73	[0.54–0.97]	*0.035*
**Quality of life expectations**						**264(199)**			
improvement							1		
deterioration							1.19	[0.81–1.74]	*0.372*
no change							1.45	[1.03–2.05]	*0.033*
					**Systemic therapy side effects**				
**Lymph node dissection(LND)**	**321(190)**					**307(176)**			
Axillary LND		1					1		
Sentinel lymph node biopsy		0.66	[0.47–0.93]	*0.019*			0.58	[0.40–0.85]	*0.005*
ALND+SLNB		0.99	[0.58–1.70]	*0.996*			0.90	[0.53–1.54]	*0.722*
No LND		0.7	[0.46–1.06]	*0.096*			0.73	[0.47–1.14]	*0.174*
**Comorbidity**									
yes		1							
no		82	[0.60–1.12]	*0.216*					
					**Constipation**				
**Lymph node dissection(LND)**	**316(138)**					322(123)			
Axillary LND		1					1		
Sentinel lymph node biopsy		0.69	[0.46–1.03]	*0.073*			0.87	[0.58–1.32]	*0.531*
ALND+SLNB		0.68	[0.35–1.32]	*0.252*			0.76	[0.38–1.51]	*0.43*
No LND		0.35	[0.19–0.66]	*0.001*			0.51	[0.28–0.94]	*0.032*
**Age (years)**									
<58							1		
> = 58							1.49	[1.02–2.16]	*0.036*
					**Dyspnea**				
**Lymph node dissection(LND)**	**325(123)**								
Axillary LND		1							
Sentinel lymph node biopsy		0.6	[0.40–0.92]	*0.019*					
ALND+SLNB		0.75	[0.39–1.45]	*0.399*					
No LND		0.49	[0.28–1.14]	*0.014*					
					**Global health**				
**Quality of life expectations**						**259(149)**			
improvement							1		
deterioration							1.60	[1.05–2.45]	*0.029*
no change							1.16	[0.77–1.74]	*0.464*
		**Financial**						**diarrhea**	
**Surgery type**	**272(58)**					283(66)			
no mastectmoy							1		
mastectomy							1.74	[1.02–2.95]	*0.039*
**Age (years)**									
<58		1							
> = 58		0.45	[0.21–0.96]	*0.041*					
**Educational level**									
low		1							
high		0.53	[0.29–0.84]	*0.032*					
					**Appetite loss**				
**Lymph node dissection(LND)**	**302(99)**								
Axillary LND		1							
Sentinel lymph node biopsy		0.45	[0.27–0.74]	*0.002*					
ALND+SLNB		0.8	[0.38–1.67]	*0.562*					
No LND		0.44	[0.20–0.96]	*0.04*					
**Quality of life expectations**	**302(99)**								
improvement		1							
deterioration		2.31	[1.38–3.88]	*0.003*					
no change		1.69	[1.00045–2.87]	*0.05*					
					**Fatigue**				
**Age(years)**						**264(189)**			
<58							1		
> = 58		NA	NA	*NA*			0.96	[0.71–1.30]	*0.809*
**social status**	**281(212)**								
Not single		1					1		
single		0.61	[0.43–0.86]	*0.005*			0.69	[0.48–0.98]	*0.043*
**Quality of life expectations**									
improvement		1							
deterioration		0.98	[0.67–1.43]	*0.944*					
no change		1.21	[0.88–1.68]	*0.235*					
					**Sexual functioning**				
	250(86)								
**social status**									
Not single		1							
single		0.41	[0.21–0.81]	*0.01*					
									
					**Pain**				
**Educational level**	283(197)								
low		1							
high		1.58	[1.17–2.14]	*0.002*					
**social status**									
Not single		1							
single		0.68	[0.47–0.99]	*0.04*					
					**Social functioning**				
**Radiotherapy**	277(168)								
yes		1							
no		0.58	[0.39–0.87]	*0.008*					
**Quality of life expectations**						**258(137)**			
improvement		1					1		
deterioration		1.45	[0.97–2.16]	*0.064*			1.75	[1.11–2.75]	*0.015*
no change		0.86	[0.58–1.27]	*0.474*			1.03	[0.66–1.60]	*0.891*
					**Physical functioning**				
**Quality of life expectations**	**311(237)**					**277(162)**			
improvement		1					1		
deterioration		2.05	[1.45–2.90]	*<0.001*			1.95	[1.33–2.86]	*0.001*
no change		1.43	[1.05–1.95	*0.023*			1.26	[0.89–1.79]	*0.188*
					**Role functioning**				
	250(86)								
**social status**									
Not single		1							
single		0.69	[0.48–0.98]	*0.041*					

**N**: number of subjects.

**NA**: not applicable (variable not included in the model).

**CI**: confidence interval.

When recalibration was taken into account for body-image, the association between TTD and the modality of surgery became non-statistically significant while radiotherapy became significantly associated with TTD. Patients who did not receive radiotherapy had a significantly longer TTD than did those who received radiotherapy: HR 0.60 [0.38–0.94].

Cox multivariate analyses showed that, expectation about QoL level at baseline was significantly associated with TTD. As example, when the recalibration component of RS was taken into account, QoL expectancy at baseline was significantly associated with TTD in GHS, physical-functioning, cognitive-functioning, social-functioning, and breast symptoms scales. Patients who expected a deterioration in their QoL at baseline had a significantly shorter TTD than patients who expected an improvement in their QoL at baseline HR: 1.60[1.05–2.45], HR: 1.95[1.33–2.86], HR: 1.64[1.07–2.52] and HR: 1.75[1.11–2.75] for GHS, physical, cognitive and social scores, respectively.

## Discussion

In this study, we examined the impact of the recalibration component of response shift on TTD estimations of QoL scores in breast cancer patients. Our results underlined that BC patients' internal standards for assessing their QoL could change during the course of treatment and disease.

The recalibration of BC patients' internal standards had a significant effect on Time to QoL score deterioration for six of the 23 dimensions. Indeed, the median TTD of the studied-population was underestimated for global health, role-functioning, social-functioning, body-image and side effects of systemic therapy when recalibration was not used as reference score to qualify QoL score deterioration. Regarding the emotional-functioning scale, the median TTD was overestimated when recalibration was not taken into account.

Our results showed that, as compared to ALND SLNB modality was independently associated with longer TTD for arm symptoms, nausea and vomiting symptoms as well as systemic therapy side effects [14], [20]–[23]. Interestingly, for breast symptoms, our results showed that SLNB followed by complementary ALND resulted in a significantly shorter TTD than for ALND alone [14]. According the surgical modality, TTD was significantly associated with diarrhea symptoms when recalibration was take into account. In contrast, for body-image, we found a significant association between the type of surgery and TTD only when the recalibration effect was not take into account. To our knowledge, no study reporting the association between the type of surgery and QoL has considered the effect of the recalibration component of RS [24]–[27]. In addition, we suggest that radiotherapy could be independently associated with a shorter time to body-image deterioration, when RS into account. These results underline the requirement to assess impact of RS through sensibility analyses.

Moreover, patients who expected deterioration or no change in their QoL level reported a significantly shorter TTD than patients who expected an improvement. Previous studies have also suggested that the high expectation of patients regarding health and QoL level, could predict better outcome [2], [28]–[31,]. Although, heterogeneity between studies clinical outcome, investigators have consistently and in a majority shown strong, statistically and clinically significant associations between patients' expectations and clinical recovery. However, the interpretation of this association remains unclear. Incorporating questions about patient expectations related to health and QoL in future trials should be promote to clarify the role for clinical outcomes.

One of the limits of our study is that we focused on the recalibration component of response-shift only using then test method.

Furthermore due to the retrospective assessment, a major limitation of the then-test method is its susceptibility to recall bias. Thereby, respondents are supposed to be able to remember their previous health and QoL level at the baseline assessment [18], [32]. The risk when using this approach could be that patients will miss to accurately recall their health and QoL level before the intervention (recall bias). Additionally, recent evidence has emerged amongst patients undertaking self-management interventions for chronic diseases that the then-test approach may contain psychometric flaws resulting from implicit theory of change, social desirability, halo effects and recall bias [33]. Including a comparison group when designing studies could help to achieve optimal use of the then-test approach. However, RS has been defined as a treatment-dependent phenomenon, pre-test, post-test and then-test scores of control subjects would only reflect effects due to history, maturation or testing. Thus, recalibration RS is only indicated if the difference between the then-test and pre-test scores are significantly larger in the experimental than in the control group [18].

Response shift has been explored over time in HRQoL through a variety of designs and statistical methods. Each of these methods is specific, with its own advantages, limitations and challenges. However, assessing response shift is of paramount importance in longitudinal HRQoL research.

In conclusion, our study showed that BC patients' internal standards change during QoL follow-up. Since patients could accommodate to the treatment toxicities or disease progression over time, this could result in the attenuation or the inflation of treatment effect estimation. Therefore, cancer clinical trials must investigate the RS effect more deeply. We encourage to plan longitudinal QoL analyses taking it into account such effect to improve interpretation of the results. Our study also showed that baseline QoL expectations were associated with QoL deterioration in several dimensions. For this reason, health care providers should give adequate counselling and psychological support to the patients at the time of the diagnosis to prevent the early QoL level deterioration.

## Supporting Information

File S1 **Tables S1 and S2.** Table S1. Univariate analyses of time to QLQ-C30 score deterioration for factors significantly affecting TTD with or without taking account of the recalibration component of RS. Table S2. Univariate analyses of time to QLQ-BR3 score deterioration for factors significantly affecting TTD with or without taking into account the recalibration component of RS.
(DOC)

## References

[1] Hagerty RG, Butow PN, Ellis PA, Lobb EA, Pendlebury S, et al (2004) Cancer patient preferences for communication of prognosis in the metastatic setting. J Clin Oncol 22(9): 1721–30. 15117995 10.1200/JCO.2004.04.095

[2] Carr AJ, Gibson B, Robinson PG (2001) Is quality of life determined by expectations or experience? BMJ 322: , 1240–1243. 10.1136/bmj.322.7296.1240 PMC1120338 11358783

[3] Allison PJ, Locker D, Feine JS (1997) Quality of life: a dynamic construct. Soc Sci Med 45(2): , 221–230. 10.1016/s0277-9536(96)00339-5 9225410

[4] Howard GS, Ralph KM, Gulanick NA, Maxwell SE, Nance SW, et al (1979) Internal invalidity in pre-test-posttest self-report evaluations and a re-evaluation of retrospective pre-tests. Appl Psychol Meas 3: 1–23.

[5] Schwartz CE, Sprangers MA (1999) Methodological approaches for assessing response shift in longitudinal health-related quality-of-life research. Soc Sci Med 48(11): 1531–1548. 10400255 10.1016/s0277-9536(99)00047-7

[6] Sprangers MA, Schwartz CE (1999) Integrating response shift into health-related quality of life research: a theoretical model. Soc Sci Med 48(11): 1507–1515. 10400253 10.1016/s0277-9536(99)00045-3

[7] Ubel PA, Peeters Y, Smith D (2010) Abandoning the language of "response shift": a plea for conceptual clarity in distinguishing scale recalibration from true changes in quality of life. Qual Life Res 19(4): 465–471. 20112000 10.1007/s11136-010-9592-x

[8] Andrykowski MA, Donovan KA, Jacobsen PB (2009) Magnitude and correlates of response shift in fatigue ratings in women undergoing adjuvant therapy for breast cancer. J Pain Symptom Manage 37(3): 341–351. 18757176 10.1016/j.jpainsymman.2008.03.01 PMC2682229

[9] Sprangers MA, Van Dam FS, Broersen J, Lodder L, Wever L, et al (1999) Revealing response shift in longitudinal research on fatigue—the use of the thentest approach. Acta Oncol 38(6): 709–718. 10522761

[10] Dabakuyo TS, Guillemin F, Conroy T, Velten M, Jolly D, et al (2013) Response shift effects on measuring post-operative quality of life among breast cancer patients: a multicenter cohort study. Qual Life Res 22: 1–11. 10.1007/s11136-012-0135-5 22383104

[11] Donaldson GW, Moinpour CM (2005) Learning to live with missing quality-of-life data in advanced-stage disease trials. J Clin Oncol 23(30): 7380–7384. 16186589 10.1200/JCO.2005.07.022

[12] Lipscomb J, Donaldson MS, Arora NK, Brown ML, Clauser SB, et al (2004) Cancer outcomes research. J Natl Cancer Inst Monogr (33): 178–197. 10.1093/jncimonographs/lgh039 15504928

[13] Bonnetain F, Dahan L, Maillard E, Ychou M, Mitry E, et al. (2010) Time until definitive quality of life score deterioration as a means of longitudinal analysis for treatment trials in patients with metastatic pancreatic adenocarcinoma. Eur J Cancer 46(15): , 2753–2762. 10.1016/j.ejca.2010.07.023 20724140

[14] Hamidou Z, Dabakuyo TS, Mercier M, Fraisse J, Causeret S, et al. (2011) Time to deterioration in quality of life score as a modality of longitudinal analysis in patients with breast cancer. Oncologist 16(10): , 1458–1468. 10.1634/theoncologist.2011-0085 PMC3228064 21948650

[15] Anota A, Hamidou Z, Paget-Bailly S, Chibaudel B, Bascoul-Mollevi C, et al.. (2013) Time to health-related quality of life score deterioration as a modality of longitudinal analysis for health-related quality of life studies in oncology: do we need RECIST for quality of life to achieve standardization?Qual Life Res DOI 10.1007/s11136-013-0583-6 10.1007/s11136-013-0583-6 PMC4282717 24277234

[16] Aaronson NK, Ahmedzai S, Bergman B, Bullinger M, Cull A, et al (1993) The European Organization for Research and Treatment of Cancer QLQ-C30: a quality-of-life instrument for use in international clinical trials in oncology. J Natl Cancer Inst 85(5): 365–376. 8433390 10.1093/jnci/85.5.365

[17] Sprangers MA, Groenvold M, Arraras JI, Franklin J, te Velde A, et al (1996) The European Organization for Research and Treatment of Cancer breast cancer-specific quality-of-life questionnaire module: first results from a three-country field study. J Clin Oncol 14(10): 2756–2768. 8874337 10.1200/JCO.1996.14.10.2756

[18] Schwartz CE, Sprangers MA (2010) Guidelines for improving the stringency of response shift research using the thentest. Qual Life Res 19(4): 455–464. 20084465 10.1007/s11136-010-9585-9

[19] Osoba D, Rodrigues G, Myles J, Zee B, Pater J (1998) Interpreting the significance of changes in health-related quality-of-life scores. J Clin Oncol 16(1): 139–44. 9440735 10.1200/JCO.1998.16.1.139

[20] Dabakuyo TS, Fraisse J, Causeret S, Gouy S, Padeano MM, et al (2009) A multicenter cohort study to compare quality of life in breast cancer patients according to sentinel lymph node biopsy or axillary lymph node dissection. Ann Oncol 20(8): 1352–61. 19468032 10.1093/annonc/mdp016

[21] Härtl K, Janni W, Kästner R, Sommer H, Strobl B, et al (2003) Impact of medical and demographic factors on long-term quality of life and body image of breast cancer patients. Ann Oncol 14(7): 1064–1071. 12853348 10.1093/annonc/mdg289

[22] Hopwood P, Haviland J, Mills J, Sumo G, Bliss M (2007) The impact of age and clinical factors on quality of life in early breast cancer: an analysis of 2208 women recruited to the UK START Trial (Standardisation of Breast Radiotherapy Trial). Breast 16(3): 241–251. 17236771 10.1016/j.breast.2006.11.003

[23] Mansel RE, Fallowfield L, Kissin M, Goyal A, Newcombe RG, et al (2006) Randomized multicenter trial of sentinel node biopsy versus standard axillary treatment in operable breast cancer: the ALMANAC Trial. J Natl Cancer Inst 98(9): 599–609. 16670385 10.1093/jnci/djj158

[24] Veronesi U, Paganelli G, Viale G, Luini A, Zurrida S, et al (2003) A randomized comparison of sentinel-node biopsy with routine axillary dissection in breast cancer. N Engl J Med 349(6): 546–553. 12904519 10.1056/NEJMoa012782

[25] Purushotham AD, Upponi S, Klevesath MB, Bobrow L, Millar K, et al (2005) Morbidity after sentinel lymph node biopsy in primary breast cancer: results from a randomized controlled trial. J Clin Oncol 23(19): 4312–4321. 15994144 10.1200/JCO.2005.03.228

[26] Arndt V, Stegmaier C, Ziegler H, Brenner H (2008) Quality of life over 5 years in women with breast cancer after breast-conserving therapy versus mastectomy: a population-based study. J Cancer Res Clin Oncol 134(12): 1311–1318. 18504613 10.1007/s00432-008-0418-y PMC12161713

[27] King MT, Kenny P, Shiell A, Hall J, Boyages J (2000) Quality of life three months and one year after first treatment for early stage breast cancer: influence of treatment and patient characteristics. Qual Life Res 9(7): 789–800. 11297021 10.1023/a:1008936830764

[28] Gepstein R, Arinzon Z, Adunsky A, Folman Y (2006) Decompression surgery for lumbar spinal stenosis in the elderly: preoperative expectations and postoperative satisfaction. Spinal Cord 44(7): 427–431. 16304562 10.1038/sj.sc.3101857

[29] Lutz GK, Butzlaff ME, Atlas SJ, Keller RB, Singer DE, et al (1999) The relation between expectations and outcomes in surgery for sciatica. J Gen Intern Med 14(12): 740–744. 10632818 10.1046/j.1525-1497.1999.10417.x PMC1496858

[30] Ronnberg K, Lind B, Zoega B, Halldin K, Gellerstedt M, et al (2007) Patients' satisfaction with provided care/information and expectations on clinical outcome after lumbar disc herniation surgery. Spine 32(2): 256–261. 17224823 10.1097/01.brs.0000251876.98496.52

[31] Myers SS, Phillips RS, Davis RB, Cherkin DC, Legedza A, et al (2007) Patient Expectations as Predictors of Outcome In Patients with Acute Low Back Pain. J Gen Intern Med 23(2): 148–53. 18066631 10.1007/s11606-007-0460-5 PMC2359167

[32] Norman G (2003) Hi! How are you? Response shift, implicit theories and differing epistemologies. Qual Life Res 12(3): 239–249. 12769136 10.1023/a:1023211129926

[33] Nolte S, Elswoth GR, Sinclair AJ, Osborne RH (2009) Tests of measurement invariance failed to support the application of the “then-test”. J Clin Epidemiol 62: 1173–80. 19595570 10.1016/j.jclinepi.2009.01.021

