# Supplementary material for: Impact of Response Shift on Time to Deterioration in Quality of Life Scores in Breast Cancer Patients
Source: PLoS One. 2014 May 14;9(5):e96848. doi: 10.1371/journal.pone.0096848 (PMC4020802; doi:10.1371/journal.pone.0096848)
Supplement: File S1 — Tables S1 and S2. Table S1. Univariate analyses of time to QLQ-C30 score deterioration for factors significantly affecting TTD with or without taking account of the recalibration component of RS. Table S2. Univariate analyses of time to QLQ-BR3 score deterioration for factors significantly affecting TTD with or without taking into account the recalibration component of RS. (DOC) [file pone.0096848.s001.doc]

Table S1: Univariate analyses of time to QLQ-C30 score deterioration for factors significantly affecting TTD with or without taking account of the recalibration component of RS

|  |  |  |  | |  |  |  | | |  |  |  |  |  |  |  |
| --- | --- | --- | --- | --- | --- | --- | --- | --- | --- | --- | --- | --- | --- | --- | --- | --- |
|  | **n** | **Event** | **median(95%CI)** | **HR** | | **(95%°CI)** | | **p** | **n** | | **Event** | **median(95%CI)** | **HR** | **(95%°CI)** | **p** | |
|  | **Without taking recalibration component of RS into account** | | |  | |  | |  |  | | **With recalibration component of RS taken into account** | | | |  | |
|  |  |  |  |  | |  | | **Global health** |  | |  |  |  |  |  | |
|  | *original scores* |  |  |  | |  | |  |  | | *then-test2* |  |  |  |  | |
| **Education Degree** |  |  |  |  | |  | |  |  | |  |  |  |  |  | |
| low | 141 | 92 | 3.2 [3.0-3.9] | 1 | |  | |  | 129 | | 66 | 6.3 [3.4-7.9] | 1 |  |  | |
| high | 142 | 101 | 0.9 [0.3-3.2] | 1.2 | | [0.90-1.59] | | *0.203* | 134 | | 84 | 3.1 [0.4-3.8] | 1.4 | [1.01-1.93] | *0.04* | |
| **Quality of life expectations** |  |  |  |  | |  | | *0.2029* |  | |  |  |  |  | *0.0221* | |
| improvement | 118 | 71 | 3.3 [3.0-6.1] | 1 | |  | |  | 107 | | 53 | 6.3 [3.3-NR] | 1 |  |  | |
| deterioration | 73 | 53 | 3.0 [0.3-3.3] | 1.32 | | [0.92-1.89] | |  | 73 | | 51 | 3 [0.3-3.4] | 1.71 | [1.16-2.52] |  | |
| no change | 128 | 90 | 3.1 [0.5-3.5] | 1.26 | | [0.92-1.73] | |  | 117 | | 64 | 6.1 [3.2-NR] | 1.18 | [0.81-1.70] |  | |
|  |  |  |  |  | |  | | **Physical functioning** |  | |  |  |  |  |  | |
|  | *original scores* |  |  |  | |  | |  |  | | *then-test3* |  |  |  |  | |
| **Chemotherapy** |  |  |  |  | |  | |  |  | |  |  |  |  |  | |
| yes | 140 | 118 | 0.5 [0.3-2.9] | 1 | |  | |  | 139 | | 113 | 0.4 [0.3-0.] | 1 |  |  | |
| no | 184 | 128 | 0.9 [0.4-3.2] | 0.73 | | [0.56-0.94] | | 0.017 | 184 | | 118 | 0.7 [0.4-3.2] | 0.73 | [0.56-0.95] | *0.022* | |
| **Age (years)** |  |  |  |  | |  | |  |  | |  |  |  |  |  | |
| <58 | 167 | 130 | 0.3 [0.2-0.5] | 1 | |  | |  | 167 | | 127 | 0.3 [0.2-0.4] | 1 |  |  | |
| >=58 | 162 | 119 | 3.0 [0.7-3.2] | 0.73 | | [0.56-0.93] | | *0.014* | 160 | | 106 | 3.0 [0.7-5.0] | 0.64 | [0.49-0.83] | *0.001* | |
| **Professional status** |  |  |  |  | |  | |  |  | |  |  |  |  |  | |
| working | 169 | 130 | 0.4 [0.2-0.6] | 1 | |  | |  | 169 | | 127 | 0.4 [0.2-0.5] | 1 |  |  | |
| not working | 149 | 112 | 2.9 [0.5-3.2] | 0.83 | | [0.64-1.07] | | *0.165* | 147 | | 99 | 1.5 [0.5-3.2] | 0.76 | [0.58-0.99] | *0.045* | |
| **Quality of life expectations** |  |  |  |  | |  | | *0.0020* |  | |  |  |  |  | *0.0434* | |
| improvement | 123 | 84 | 3.1 [0.6-3.4] | 1 | |  | |  | 121 | | 80 | 3.1 [0.5-3.3] | 1 |  |  | |
| deterioration | 73 | 65 | 0 .3 [0.2-0.4] | 1.81 | | [1.31-2.52] | |  | 73 | | 60 | 0.3 [0.2-0.4] | 1.54 | [1.10-2.16] |  | |
| no change | 129 | 98 | 0.5 [0.3-2.9] | 1.26 | | [0.94-1.68] | |  | 129 | | 91 | 0.5 [0.3-1.8] | 1.14 | [0.84-1.54] |  | |

Table S1 :( continued)

|  |  |  |  |  |  |  |  |  |  |  |  |  |
| --- | --- | --- | --- | --- | --- | --- | --- | --- | --- | --- | --- | --- |
|  | **n** | **Event** | **median(95%CI)** | **HR** | **(95%°CI)** | **p** | **n** | **Event** | **median(95%CI)** | **HR** | **(95%°CI)** | **p** |
|  | **Without taking recalibration component of RS into account** | | |  |  |  |  | **With recalibration component of RS taken into account** | | | |  |
|  |  |  |  |  |  | **Role functioning** |  |  |  |  |  |  |
|  | *original scores* |  |  |  |  |  |  | *then-test2&then-test3* | |  |  |  |
| **Stage(AJCC)** |  |  |  |  |  | *0.8434* |  |  |  |  |  | *0.0403* |
| 0 | 69 | 43 | 3.1 [0.4-4.0] | 1 |  |  | 65 | 43 | 2.9 [0.3-3.3] | 1 |  |  |
| 1 | 114 | 84 | 3.2 [3.0-3.4] | 1.04 | [.722-1.50] |  | 107 | 61 | 6.3 [3.4-6.6] | 0.56 | [0.38-0.83] |  |
| 2 | 100 | 72 | 3.2 [3.0-3.6] | 1.08 | [0.74-1.58] |  | 96 | 64 | 6 [3.2-6.3] | 0.75 | [0.51-1.11] |  |
| 3-4 | 14 | 9 | 4.0 [0.3-NR] | 0.80 | [0.39-1.66] |  | 14 | 7 | 6.2 [0.5-NR] | 0.64 | [0.29-1.44] |  |
| **Professional status** |  |  |  |  |  |  |  |  |  |  |  |  |
| working | 168 | 119 | 3.1 [0.8-3.3] | 1 |  |  | 157 | 104 | 3.1 [0.6-6.0] | 1 |  |  |
| not working | 144 | 99 | 3.3 [3.1-3.5] | 0.86 | [0.66-1.13] | *0.294* | 139 | 80 | 6.2 [3.7-6.5] | 0.71 | [0.53-0.96] | *0.026* |
| **Age** |  |  |  |  |  |  |  |  |  |  |  |  |
| <58 | 167 | 119 | 3.1 [1.8-3.3] | 1 |  |  | 156 | 101 | 3.3 [0.7-6.1] | 1 |  |  |
| >=58 | 157 | 107 | 3.3 [3.1-3.6] | 0.85 | [0.65-1.10] | *0.236* | 152 | 90 | 6.1[3.4-6.5] | 0.73 | [0.55-0.97] | *0.034* |
| **Quality of life expectations** |  |  |  |  |  | *0.3281* |  |  |  |  |  | *0.1589* |
| improvement | 120 | 76 | 3.3 [1.9-3.7] | 1 |  |  | 109 | 62 | 3.7 [3.0-6.5] | 1 |  |  |
| deterioration | 72 | 58 | 3.2 [0.5-3.4] | 0.22 | [1.82-] |  | 73 | 53 | 3.4 [0.5-6.0] | 1.33 | [0.92-1.92] |  |
| no change | 128 | 90 | 3.1 [3.0-3.3] | 1.08 | [0.79-1.47] |  | 121 | 72 | 6.2 [3.2-6.5] | 0.95 | [0.67-1.33] |  |
|  |  |  |  |  |  | **Emotional functioning** |  |  |  |  |  |  |
|  | *original scores* |  |  |  |  |  |  | *then-test2& then-test3* | |  |  |  |
| **Chemotherapy** |  |  |  |  |  |  |  |  |  |  |  |  |
| yes | 141 | 73 | 6.3 [3.5-7.2] | 1 |  |  | 132 | 83 | 3.2 [3.0-3.7] | 1 |  |  |
| no | 184 | 79 | 6.3 [6.4-NR] | 0.77 | [0 .56-1.07] | *0.124* | 173 | 88 | 6.4 [3.2-12.2] | 0.72 | [0.53-0.98] | *0.039* |
| **Hormone therapy** |  |  |  |  |  |  |  |  |  |  |  |  |
| yes | 152 | 77 | 6.4 [ 3.7-7.5] | 1 |  |  | 141 | 91 | 3.2 [3.0-3.7] | 1 |  |  |
| no | 174 | 75 | 6.8 [6.4-NR] | 0 .79 | [0 .57-1.09] | *0.158* | 165 | 82 | 6.4 [3.4-6.9] | 0.72 | [0.53-0.97] | *0.034* |

Table S1 :( continued)

|  | **n** | **Event** | **median(95%CI)** | **HR** | **(95%°CI)** | **p** | **n** | **Event** | **median(95%CI)** | **HR** | **(95%°CI)** | **p** |
| --- | --- | --- | --- | --- | --- | --- | --- | --- | --- | --- | --- | --- |
|  | **Without taking recalibration component of RS into account** | | |  |  |  |  | **With recalibration component of RS taken into account** | | | |  |
|  |  |  |  |  |  | **Cognitive functioning** |  |  |  |  |  |  |
| **Quality of life expectations** |  |  |  |  |  | *0.2187* |  |  |  |  |  | *0.0399* |
| improvement | 122 | 56 | 7.5 [5.7-8.6] | 1 |  |  | 109 | 44 | NR [6.1-NR] | 1 |  |  |
| deterioration | 72 | 41 | 3.5 [3.2-NR] | 1.31 | [0.87-1.97] |  | 74 | 43 | 3.6 [3.0-6.4] | 1.64 | [1.07-2.50] |  |
| no change | 130 | 56 | 7.1 [6.1-NR | 0.92 | [0.63-1.33] |  | 118 | 50 | 6.8 [6.4-NR] | 1.02 | [0.68-1.53] |  |
|  |  |  |  |  |  |  |  |  |  |  |  |  |
|  |  |  |  |  |  | **Social functioning** |  |  |  |  |  |  |
|  | *original scores* |  |  |  |  |  |  | *then-test2& then-test3* | |  |  |  |
| **Chemotherapy** |  |  |  |  |  |  |  |  |  |  |  |  |
| yes | 136 | 98 | 3.2 [3.0-3.5] | 1 |  |  | 129 | 82 | 5.7 [3.2-6.2] | 1 |  |  |
| no | 184 | 91 | 6.3 [3.5-NR] | 0.55 | [0.41-0.73] | *<0.0001* | 173 | 71 | 7.8 [6.6-NR] | 0.56 | [0.40-0.77] | *<0.0001* |
| **radiotherapy** |  |  |  |  |  |  |  |  |  |  |  |  |
| yes | 216 | 143 | 3.4 [3.2-3.8] | 1 |  |  | 208 | 106 | 6.2 [6.0-7.2] | 1 |  |  |
| no | 104 | 46 | 6.5 [4.7-NR] | 0.61 | [0.43-0.85] | *0.004* | 94 | 47 | 6.5 [3.4-6.9] | 1.04 | [0.73-1.47] | *0.801* |
| **Age(years)** |  |  |  |  |  |  |  |  |  |  |  |  |
| <58 | 164 | 98 | 3.4 [3.1-6.1] | 1 |  |  | 153 | 85 | 5.7 [3.1-6.6] | 1 |  |  |
| >=58 | 161 | 92 | 4.3 [3.4-6.5] | 0.79 | [0.59-1.05] | *0.114* | 152 | 69 | 6.6 [6.2-NR] | 0.68 | [0.49-0.94] | *0.021* |
| **Professional status** |  |  |  |  |  |  |  |  |  |  |  |  |
| working | 166 | 100 | 3.3 [3.1-6.1] | 1 |  |  | 155 | 94 | 3.6 [3.1-6.2] | 1 |  |  |
| not working | 148 | 86 | 4.2 [3.4-6.2] | 0.84 | [0.63-1.12] | *0.244* | 138 | 57 | 6.7 [6.3-NR] | 0.57 | [0.41-0.79] | *0.001* |
| **Quality of life expectations** |  |  |  |  |  | *0.0022* |  |  |  |  |  | *0.0120* |
| improvement | 124 | 71 | 4.2 [3.4-6.2] | 1 |  |  | 52 | 108 | 6.3 [0.1-6.1] | 1 |  |  |
| deterioration | 72 | 55 | 3.1 [0.5-3.4] | 1.57 | [1.10-2.24] |  | 47 | 73 | 3.6 [0.7-0.5] | 1.63 | [1.09-2.43] |  |
| no change | 125 | 63 | 6.3 [3.2-NR] | 0.818 | [.583-1.14] |  | 52 | 118 | 6.7 [0.1-6.1] | 0.91 | [0.62-1.34] |  |

Table S1 :( continued)

|  | **n** | **Event** | **median(95%CI)** | **HR** | **(95%°CI)** | **p** | **n** | **Event** | **median(95%CI)** | **HR** | **(95%°CI)** | **p** |
| --- | --- | --- | --- | --- | --- | --- | --- | --- | --- | --- | --- | --- |
|  | **Without taking recalibration component of RS into account** | | |  |  |  |  | **With recalibration component of RS taken into account** | | | |  |
|  |  |  |  |  |  | **Nausea** |  |  |  |  |  |  |
|  | *original scores* |  |  |  |  |  |  | *then-test3* |  |  |  |  |
| **Lymph node dissection(LND)** |  |  |  |  |  | *0.0016* |  |  |  |  |  | *0.0202* |
| Axillary LND | 118 | 57 | 6.3 [5.1-NR] | 1 |  |  | 118 | 43 | 7.0 [6.3-NR] | 1 |  |  |
| Sentinel lymph node biopsy | 115 | 29 | 8.2 [NR-NR] | 0.42 | [0.27-0.66] |  | 114 | 23 | NR [NR-NR] | 0.48 | [0.29-0.80] |  |
| ALND+SLNB | 30 | 13 | 6.2 [3.6-NR] | 0.80 | [0.43-1.46] |  | 30 | 7 | 12.2 [[6.5-NR] | 0.45 | [0.20-1.03] |  |
| No LND | 65 | 21 | NR [6.3-NR] | 0.61 | [0.37-1.01] |  | 65 | 17 | NR [6.5-NR] | 0.70 | [0.39-1.23] |  |
| **Chemotherapy** |  |  |  |  |  |  |  |  |  |  |  |  |
| yes | 141 | 74 | 6.2 [4.0-6.5] | 1 |  |  | 142 | 58 | 6.6 [6.3-NR] | 1 |  |  |
| no | 185 | 46 | 8.2 [8.2-NR] | 0.39 | [0.26-0.56] | *<0.0001* | 183 | 32 | 12.2 [12.2-NR] | 0.38 | [0.24-0.59] | *<0.0001* |
| **Age(years)** |  |  |  |  |  |  |  |  |  |  |  |  |
| <58 | 168 | 72 | 6.5 [6.3-NR] | 1 |  |  | 168 | 52 | 12.2 [6.6-NR] | 1 |  |  |
| >=58 | 163 | 49 | 8.2 [NR-NR] | 0.60 | [0.42-0.87] | *0.007* | 161 | 38 | NR [NR-NR] | 0.67 | [0.44-1.02] | *0.064* |
| **Stage(AJCC)** |  |  |  |  |  | *0.0196* |  |  |  |  |  | *0.1396* |
| 0 | 70 | 19 | NR [6.5-NR] | 1 |  |  | 70 | 14 | NR [6.6-NR] | 1 |  |  |
| 1 | 115 | 37 | 8.2 [7-NR] | 1.09 | [0.63-1.91] |  | 113 | 29 | 12.2 [NR-NR] | 1.11 | [0.59-2.12] |  |
| 2 | 102 | 47 | 6.3 [5.7-NR] | 1.87 | [1.09-3.19] |  | 103 | 34 | 7 [6.4-NR] | 1.68 | [0.90-3.15] |  |
| 3-4 | 14 | 7 | 3.4 [2.9-NR] | 2.40 | [1.00-5.73] |  | 14 | 6 | 6.3 [2.5-NR] | 2.34 | [0.89-6.13] |  |
| **social status** |  |  |  |  |  |  |  |  |  |  |  |  |
| couple | 245 | 91 | 8.2 [6.6-NR] | 1 |  |  | 244 | 74 | 12.2 [6.9-NR] | 1 |  |  |
| single | 79 | 28 | NR [6.2-NR] | 0.95 | [0.62-1.46] | *0.84* | 78 | 14 | NR [6.7-NR] | 0.55 | [0.31-0.97] | *0.042* |
| **Quality of life expectations** |  |  |  |  |  | *0.0105* |  |  |  |  |  | *0.0763* |
| improvement | 125 | 43 | NR [6.5-NR] | 1 |  |  | 123 | 30 | NR [NR-NR] | 1 |  |  |
| deterioration | 72 | 39 | 6.3 [4.2-NR] | 1.58 | [1.02-2.44] |  | 72 | 29 | 7.0 [6.3-NR] | 1.55 | [0.93-2.59] |  |
| no change | 130 | 38 | NR [NR-NR] | 0.78 | [0.51-1.22] |  | 130 | 30 | NR [6.9-NR] | 0.86 | [0.51-1.43] |  |

Table S1 :( continued)

|  | **n** | **Event** | **median(95%CI)** | **HR** | **(95%°CI)** | **p** | **n** | **Event** | **median(95%CI)** | **HR** | **(95%°CI)** | **p** |
| --- | --- | --- | --- | --- | --- | --- | --- | --- | --- | --- | --- | --- |
|  | **Without taking recalibration component of RS into account** | | |  |  |  |  | **With recalibration component of RS taken into account** | | |  |  |
|  | *original scores* |  |  |  |  | **Fatigue** |  | *then-test1& then-test3* | |  |  |  |
| **social status** |  |  |  |  |  |  |  |  |  |  |  |  |
| couple | 240 | 188 | 1 [0.5-3.06] | 1 |  |  | 228 | 167 | 0.8 [0.4-3.0] | 1 |  |  |
| single | 78 | 51 | 3.3 [0.7-4.7] | 0.72 | [0.52-0.98] | *0.04* | 73 | 46 | 3.4 [0.9-6.3] | 0.72 | [0.52-1.00] | *0.054* |
| **Education Degree** |  |  |  |  |  |  |  |  |  |  |  |  |
| low | 140 | 101 | 3.0 [0.7-3.3] | 1 |  |  | 137 | 93 | 3.2 [1.5-3.7] | 1 |  |  |
| high | 142 | 112 | 0.5 [0.3-3.0] | 1.24 | [0.95-1.63] | *0.111* | 131 | 98 | 0.4 [0.3-3.0] | 1.37 | [1.03-1.82] | *0.03* |
| **Professional status** |  |  |  |  |  |  |  |  |  |  |  |  |
| working | 166 | 125 | 0.5 [0.3-3.0] | 1 |  |  | 155 | 114 | 0.5 [0.3-3.0] | 1 |  |  |
| not working | 147 | 110 | 3.1 [1.5-3.3] | 0.86 | [0.67-1.12] | *0.275* | 141 | 96 | 3.2 [1.5-3.7] | 0.75 | [0.57-0.99] | *0.045* |
|  | *original scores* |  |  |  |  | **Pain** |  | *then-test3* |  |  |  |  |
| **Age (years)** |  |  |  |  |  |  |  |  |  |  |  |  |
| <58 | 167 | 128 | 0.6 [0.4-3.0] | 1 |  |  | 166 | 118 | 0.5 [0.3-1.8] | 1 |  |  |
| >=58 | 164 | 102 | 3.4 [3.1-4.7] | 0.64 | [0.49-0.83] | *0.001* | 164 | 99 | 3.3 [3.0-6.0] | 0.63 | [0.48-0.82] | *0.001* |
| **Comorbidity** |  |  |  |  |  |  |  |  |  |  |  |  |
| yes | 207 | 134 | 3.3 [1.9-3.6] | 1 |  |  | 208 | 131 | 3.2 [1.8-3.4] | 1 |  |  |
| no | 123 | 96 | 0.6 [0.4-3.1] | 1.42 | [1.09-1.84] | *0.009* | 121 | 86 | 0.5 [0.3-3.1] | 1.28 | [0.98-1.69] | *0.069* |
| **social status** |  |  |  |  |  |  |  |  |  |  |  |  |
| couple | 2545 | 179 | 3.0 [0.5-3.2] | 1 |  |  | 245 | 169 | 1.8 [0.5-3.1] | 1 |  |  |
| single | 80 | 47 | 3.4 [0.9-7] | 0.69 | [0.50-0.96] | *0.028* | 79 | 45 | 3.7 [0.9-6.6] | 0.72 | [0.52-1.01] | *0.059* |
| **Education Degree** |  |  |  |  |  |  |  |  |  |  |  |  |
| low | 145 | 86 | 3.7 [3.1-6.2] | 1 |  |  | 144 | 85 | 3.3 [3.0-5.7] | 1 |  |  |
| high | 144 | 113 | 0.5 [0.3-3.0] | 1.75 | [1.32-2.33] | *<0.0001* | 143 | 108 | 0.5 [0.3-1.8] | 1.63 | [1.23-2.17] | *0.001* |
| **Professional status** |  |  |  |  |  |  |  |  |  |  |  |  |
| working | 169 | 128 | 0.5 [0.4-1.9] | 1 |  |  | 168 | 123 | 0.5 [0.4-0.83] | 1 |  |  |
| not working | 151 | 96 | 3.4 [3.2-5.7] | 0.66 | [0.50-0.86] | *0.003* | 151 | 89 | 3.4 [3.1-6.2] | 0.61 | [0.46-0.80] | *<0.0001* |

Table S1 :( continued)

|  | **n** | **Event** | **median(95%CI)** | **HR** | **(95%°CI)** | **p** | **n** | **Event** | **median(95%CI)** | **HR** | **(95%°CI)** | **p** |
| --- | --- | --- | --- | --- | --- | --- | --- | --- | --- | --- | --- | --- |
|  | **Without taking recalibration component of RS into account** | | |  |  |  |  | **With recalibration component of RS taken into account** | | | |  |
|  |  |  |  |  |  | **Dyspnea** |  |  |  |  |  |  |
|  | *original scores* |  |  |  |  |  |  | *then-test1& then-test3* | |  |  |  |
| **Lymph node dissection(LND)** |  |  |  |  |  | *0.0502* |  |  |  |  |  | *0.1401* |
| Axillary LND | 117 | 55 | 6.3 [6.0-8.0] | 1 |  |  | 109 | 46 | 6.9 [6.3-7.9] | 1 |  |  |
| Sentinel lymph node biopsy | 114 | 40 | 7.2 [6.5-NR] | 0.66 | [0.44-1.00] |  | 108 | 35 | 7.2 [6.8-NR] | 0.74 | [0.47-1.15] |  |
| ALND+SLNB | 30 | 12 | 7.5 [3.6-NR] | 0.73 | [0.39-1.37] |  | 27 | 12 | 7.1 [6.1-NR] | 0.80 | [0.41-1.55] |  |
| No LND | 64 | 16 | NR [7.2-NR] | 0.49 | [0.28-0.86] |  | 62 | 13 | NR [6.6-NR] | 0.50 | [0.27-0.93] |  |
| **Chemotherapy** |  |  |  |  |  |  |  |  |  |  |  |  |
| yes | 141 | 67 | 6.3 [6.0-8.0] | 1 |  |  | 132 | 60 | 6.6 [6.3-6.9] | 1 |  |  |
| no | 181 | 55 | 7.2 [7.1-NR] | 0.52 | [0.36-0.75] | *<0.0001* | 173 | 45 | 7.2 [7.1-.] | 0.53 | [0.36-0.79] | *0.002* |
| **Stage(AJCC)** |  |  |  |  |  | *0.2123* |  |  |  |  |  | *0.0465* |
| 0 | 70 | 22 | 7.2 [6.4-NR] | 1 |  |  | 67 | 13 | NR [6.6-NR] | 1 |  |  |
| 1 | 114 | 44 | 7.2 [6.4-NR] | 1.15 | [0.69-1.92] |  | 107 | 39 | 7.1 [6.6-8.1] | 1.69 | [0.90-3.17] |  |
| 2 | 101 | 43 | 7.5 [6.0-NR] | 1.50 | [0.90-2.51] |  | 96 | 41 | 6.9 [6.3-7.5] | 2.31 | [1.23-4.32] |  |
| 3-4 | 14 | 3 | 7.2 [6.3-NR] | 0.62 | [0.18-2.08] |  | 12 | 3 | NR [2.8-NR] | 1.44 | [0.41-5.08] |  |
|  |  |  |  |  |  | **Insomnia** |  |  |  |  |  |  |
|  | *original scores* |  |  |  |  |  |  | *then-test2& then-test3* | |  |  |  |
| **Hormone therapy** |  |  |  |  |  |  |  |  |  |  |  |  |
| yes | 151 | 66 | 6.8 [5.9-NR] | 1 |  |  | 138 | 57 | 6.8 [6.4-NR] | 1 |  |  |
| no | 172 | 72 | 7.8 [6.4-8.6] | 0.91 | [0.65-1.27] | *0.604* | 161 | 83 | 6.5 [3.2-6.7] | 1.43 | [1.02-2.01] | *0.036* |

Table S1 :( continued)

|  | **n** | **Event** | **median(95%CI)** | **HR** | **(95%°CI)** | **p** | **n** | **Event** | **median(95%CI)** | **HR** | **(95%°CI)** | **p** |
| --- | --- | --- | --- | --- | --- | --- | --- | --- | --- | --- | --- | --- |
|  | **Without taking recalibration component of RS into account** | | |  |  |  |  | **With recalibration component of RS taken into account** | | | |  |
|  |  |  |  |  |  | **Appetite loss** |  |  |  |  |  |  |
|  | *original scores* |  |  |  |  |  |  | *then-test1&then-test3* | |  |  |  |
| **Lymph node dissection(LND)** |  |  |  |  |  | *0.0009* |  |  |  |  |  | *0.2829* |
| Axillary LND | 118 | 52 | 6.8 [4.0-NR] | 1 |  |  | 109 | 39 | 6.9 [6.5-NR] | 1 |  |  |
| Sentinel lymph node biopsy | 116 | 29 | 8.6 [7.3-NR] | 0.49 | [0.31-0.78] |  | 108 | 28 | 8.6 [7.3-NR] | 0.68 | [0.42-1.11] |  |
| ALND+SLNB | 30 | 13 | NR [3.5-NR] | 0.92 | [0.50-1.70] |  | 27 | 8 | NR [6.1-NR | 0.72 | [0.33-1.56] |  |
| No LND | 63 | 11 | NR [NR-NR] | 0.36 | [0.18-0.69] |  | 61 | 12 | NR [6.6-NR] | 0.58 | [0.30-1.12] |  |
| **Chemotherapy** |  |  |  |  |  |  |  |  |  |  |  |  |
| yes | 142 | 68 | 6.6 [3.8-NR] | 1 |  |  | 131 | 50 | 6.8 [6.4-NR] | 1 |  |  |
| no | 183 | 36 | NR [8.6-NR] | 0.35 | [0.23-0.52] | *<0.0001* | 173 | 36 | NR [8.6-NR] | 0.52 | [0.34-0.81] | *0.004* |
| **Stage(AJCC)** |  |  |  |  |  | *<0.0001* |  |  |  |  |  | *0.0015* |
| 0 | 68 | 10 | NR [NR-NR] | 1 |  |  | 66 | 9 | NR [NR-NR] | 1 |  |  |
| 1 | 115 | 30 | 8.6 [8.6-NR] | 1.80 | [0.88-3.70] |  | 107 | 27 | 8.6 [8.6-NR] | 1.62 | [0.76-3.45] |  |
| 2 | 104 | 52 | 6.3 [3.5-NR] | 3.98 | [2.02-7.84] |  | 96 | 40 | 6.8 [6.2-NR] | 3.01 | [1.46-6.22] |  |
| 3-4 | 13 | 7 | 6.3 [0.6-NR] | 4.66 | [1.77-12.2] |  | 12 | 6 | 6.3 [0.3-NR] | 4.38 | [1.55-12.3] |  |
| **Quality of life expectations** |  |  |  |  |  | *0.0034* |  |  |  |  |  | *0.0998* |
| improvement | 122 | 29 | 8.6 [8.6-NR] | 1 |  |  | 115 | 26 | 8.6 [8.6-NR] | 1 |  |  |
| deterioration | 73 | 37 | 6.3 [3.6-NR] | 2.26 | [1.39-3.68] |  | 66 | 27 | 6.8 [6.2-NR] | 1.79 | [1.04-3.07] |  |
| no change | 131 | 39 | NR [NR-NR] | 1.25 | [0.77-2.03] |  | 120 | 33 | NR [6.6-NR] | 1.18 | [0.70-1.97] |  |

Table S1 :( continued)

|  | **n** | **Event** | **median(95%CI)** | **HR** | **(95%°CI)** | **p** | **n** | **Event** | **median(95%CI)** | **HR** | **(95%°CI)** | **p** |
| --- | --- | --- | --- | --- | --- | --- | --- | --- | --- | --- | --- | --- |
|  | **Without taking recalibration component of RS into account** | | |  |  |  |  | **With recalibration component of RS taken into account** | | | |  |
|  |  |  |  |  |  | **Constipation** |  |  |  |  |  |  |
|  | *original scores* |  |  |  |  |  |  | *then-test3* |  |  |  |  |
| **Lymph node dissection(LND)** |  |  |  |  |  | *0.0020* |  |  |  |  |  | *0.0776* |
| Axillary LND | 118 | 65 | 6.0 [3.3-NR] | 1 |  |  | 117 | 52 | 6.9 [6.0-NR] | 1 |  |  |
| Sentinel lymph node biopsy | 112 | 48 | 7.8 [6.3-NR] | 0.7 | [0.50-1.07] |  | 112 | 46 | 7.8 [6.3-NR] | 0.90 | [0.61-1.35] |  |
| ALND+SLNB | 30 | 14 | 6.2 [3.1-NR] | 0.8 | [0.45-1.44] |  | 30 | 11 | NR [3.7-NR] | 0.77 | [0.40-1.49] |  |
| No LND | 64 | 15 | NR [NR-NR] | 0.3 | [0.20-0.64] |  | 66 | 15 | NR [NR-NR] | 0.49 | [0.27-0.88] |  |
| **Chemotherapy** |  |  |  |  |  |  |  |  |  |  |  |  |
| yes | 138 | 75 | 5.9 [3.2-NR] | 1 |  |  | 138 | 64 | 6.6 [6.0-NR] | 1 |  |  |
| no | 184 | 66 | 9.5 [6.8-NR] | 0.5 | [0.40-0.79] | *0.001* | 185 | 59 | 8.2 [8.2-NR] | 0.64 | [0.45-0.91] | *0.015* |
| **Stage(AJCC)** |  |  |  |  |  | *0.0096* |  |  |  |  |  | *0.1792* |
| 0 | 70 | 19 | NR [NR-NR] | 1 |  |  | 72 | 19 | NR [NR-NR] | 1 |  |  |
| 1 | 114 | 51 | 7.8 [6.1-NR] | 1.7 | [1.00-2.89] |  | 112 | 45 | 7.8 [6.3-NR] | 1.42 | [0.83-2.43] |  |
| 2 | 100 | 55 | 5.9 [3.5-NR] | 2.3 | [1.38-3.94] |  | 101 | 46 | 6.6 [6.0-NR] | 1.78 | [1.04-3.05] |  |
| 3-4 | 14 | 6 | 6.3 [0.3-NR] | 1.7 | [0.68-4.27] |  | 14 | 5 | NR [0.3-NR] | 1.34 | [0.50-3.60] |  |
| **Quality of life expectations** |  |  |  |  |  | *0.0651* |  |  |  |  |  | *0.2940* |
| improvement | 121 | 43 | 7.8 [6.5-NR] | 1 |  |  | 121 | 39 | 7.9 [7.9-NR] | 1 |  |  |
| detrioration | 74 | 39 | 6.3 [3.2-9.5] | 1.6 | [1.06-2.52] |  | 74 | 33 | 7.8 [6.0-NR] | 1.41 | [0.89-2.25] |  |
| no change | 128 | 58 | 6.8 [6.0-NR] | 1.4 | [0.94-2.08] |  | 127 | 50 | NR [6.4-NR] | 1.28 | [0.84-1.95] |  |
|  |  |  |  |  |  | **Diarrhea** |  |  |  |  |  |  |
|  | *original scores* |  |  |  |  |  |  | *then-test2&then-test3* | |  |  |  |
| **Surgery type** |  |  |  |  |  |  |  |  |  |  |  |  |
| no mastectmoy | 203 | 29 | NR [8.2-NR] | 1 |  |  | 192 | 34 | NR [8.2-NR] | 1 |  |  |
| mastectomy | 112 | 28 | NR [NR-NR] | 1.7 | [1.07-3.02] | *0.027* | 105 | 33 | NR [6.6-NR] | 1.88 | [1.16-3.04] | *0.01* |
|  |  |  |  |  |  |  |  |  |  |  |  |  |

Table S2: Univariate analyses of time to QLQ-BR3 score deterioration for factors significantly affecting TTD with or without taking into account the recalibration component of RS

|  | **n** | **Event** | **median(95%CI)** | **HR** | **(95%°CI)** | ***p*** | **n** | **Event** | **median(95%CI)** | **HR** | **(95%°CI)** | ***p*** |
| --- | --- | --- | --- | --- | --- | --- | --- | --- | --- | --- | --- | --- |
|  | **Without taking recalibration component of RS into account** | | | |  |  |  | **With recalibration component of RS taken into account** | | | |  |
|  |  |  |  |  |  | **Body image** |  |  |  |  |  |  |
|  | *original scores* |  |  |  |  |  |  | *then-test2&then-test3* | |  |  |  |
| **Lymph node dissection(LND)** |  |  |  |  |  | *0.0122* |  |  |  |  |  | *0.0038* |
| Axillary LND | 110 | 81 | 3.1 [2.9-3.3] | 1 |  |  | 109 | 68 | 3.5 [3.1-6.2] | 1 |  |  |
| Sentinel lymph node biopsy | 108 | 77 | 3.1 [3.0-3.5] | 0.93 | [0.68-1.27] |  | 106 | 54 | 6.3 [6.0-6.7] | 0.65 | [0.45-0.93] |  |
| ALND+SLNB | 27 | 17 | 3.6 [3.0-6.2] | 0.77 | [0.46-1.31] |  | 26 | 12 | 6.5 [2.2-NR] | 0.57 | [0.30-1.05] |  |
| No LND | 60 | 28 | 6.4 [3.3-NR] | 0.51 | [0.33-0.79] |  | 59 | 20 | NR [6.1-NR] | 0.44 | [0.26-0.72] |  |
| **Surgery type** |  |  |  |  |  |  |  |  |  |  |  |  |
| no mastectmoy | 188 | 112 | 3.7 [3.3-6.2] | 1 |  |  | 190 | 90 | 6.3 [6.1-6.8] | 1 |  |  |
| mastectomy | 108 | 87 | 1.9 [0.5-3.2] | 1.88 | [1.42-2.50] | *<0.0001* | 99 | 61 | 6.0 [3.1-6.3] | 1.37 | [0.99-1.89] | *0.057* |
| **Chemotherapy** |  |  |  |  |  |  |  |  |  |  |  |  |
| yes | 132 | 103 | 3.1 [2.9-3.2] | 1 |  |  | 130 | 87 | 5.8 [3.2-6.2] | 1 |  |  |
| no | 170 | 97 | 3.6 [3.3-6.1] | 0.6 | [0.45-0.79] | *<0.0001* | 168 | 65 | NR [6.5-NR] | 0.47 | [0.34-0.65] | *<0.0001* |
| **Radiotherapy** |  |  |  |  |  |  |  |  |  |  |  |  |
| yes | 207 | 139 | 3.3 [3.1-3.5] | 1 |  |  | 207 | 115 | 6.1 [3.4-6.3] | 1 |  |  |
| no | 95 | 61 | 3.3 [3.0-3.6] | 0.99 | [0.73-1.33] | *0.951* | 91 | 38 | 6.5 [6.3-NR] | 0.66 | [0.45-0.95] | *0.029* |
| **Comorbidity** |  |  |  |  |  |  |  |  |  |  |  |  |
| yes | 190 | 136 | 3.2 [3.1-3.4] | 1 |  |  | 188 | 95 | 6.3 [6.1-6.6] | 1 |  |  |
| no | 116 | 68 | 3.6 [3.1-6.3] | 0.73 | [0.54-0.98] | *0.038* | 112 | 59 | 6.1 [3.1-6.7] | 1.15 | [0.83-1.59] | *0.385* |
| **Stage(AJCC)** |  |  |  |  |  | *0.1033* |  |  |  |  |  | *0.0065* |
| 0 | 66 | 35 | 3.8 [3.2-NR] | 1 |  |  | 64 | 26 | 6.6 [3.8-NR] | 1 |  |  |
| 1 | 106 | 75 | 3.2 [3.0-3.5] | 1.44 | [0.96-2.15] |  | 105 | 55 | 6.2 [3.6-6.7] | 1.23 | [0.77-1.96] |  |
| 2 | 97 | 70 | 3.2 [0.7-3.4] | 1.61 | [1.07-2.42] |  | 94 | 55 | 6.0 [3.4-6.3] | 1.55 | [0.97-2.48] |  |
| 3-4 | 11 | 8 | 0.6 [0.2-NR] | 1.73 | [0.80-3.75] |  | 12 | 10 | 0.3 [0.1-0.6] | 3.96 | [1.89-8.28] |  |
| **Quality of life expectations** |  |  |  |  |  | *0.0816* |  |  |  |  |  | *0.0076* |
| improvement | 114 | 73 | 3.5 [3.1-6.1] | 1 |  |  | 107 | 57 | 6.2 [3.3-6.5] | 1 |  |  |
| detrioration | 68 | 53 | 3.0 [0.46-3.3] | 1.44 | [1.01-2.06] |  | 72 | 46 | 6.0 [2.2-6.2] | 1.34 | [0.91-1.98] |  |
| no change | 123 | 77 | 3.3 [3.1-3.9] | 1 | [0.72-1.38] |  | 117 | 49 | 6.6 [6.3-NR] | 0.7 | [0.48-1.03] |  |

Table S2: (continued)

|  | **n** | **Event** | **median(95%CI)** | **HR** | **(95%°CI)** | ***p*** | **n** | **Event** | **median(95%CI)** | **HR** | **(95%°CI)** | ***p*** |
| --- | --- | --- | --- | --- | --- | --- | --- | --- | --- | --- | --- | --- |
|  |  | **Without taking recalibration component of RS into account** | | | |  |  | **With recalibration component of RS taken into account** | | | |  |
|  |  |  |  |  |  | **Sexual functioning** |  |  |  |  |  |  |
| **Age(years)** | *original scores* |  |  |  |  |  |  | *then-test3* |  |  |  |  |
| <58 | 149 | 55 | NR [6.5-NR] | 1 |  |  | 146 | 56 | 7.2 [6.1-NR] | 1 |  |  |
| >=58 | 135 | 40 | 8.6 [NR-NR] | 0.76 | [0.50-1.14] | *0.192* | 131 | 34 | 9.8 [6.9-NR] | 0.58 | [0.37-0.89] | *0.013* |
| **social status** |  |  |  |  |  |  |  |  |  |  |  |  |
| couple | 217 | 82 | 8.6 [7.2-NR] | 1 |  |  | 213 | 77 | 9.8 [6.5-NR] | 1 |  |  |
| single | 63 | 13 | NR [NR-NR] | 0.52 | [0.29-0.94] | *0.03* | 60 | 13 | NR [6.9-NR] | 0.57 | [0.31-1.02] | *0.062* |
|  |  |  |  |  |  | **Sexual enjoyment** |  |  |  |  |  |  |
|  | *original scores* |  |  |  |  |  |  | *then-test2& then-test3* | |  |  |  |
| **Surgery type** |  |  |  |  |  |  |  |  |  |  |  |  |
| no mastectmoy | 85 | 33 | 6.7 [6.4-NR] | 1 |  |  | 105 | 38 | NR [6.4-NR] | 1 |  |  |
| mastectomy | 38 | 22 | 3.5 [3.0-NR] | 1.94 | [1.12-3.36] | *0.018* | 39 | 15 | 9.56 [3.5-NR] | 1.1 | [0.62-1.96] | *0.724* |
| **Stage(AJCC)** |  |  |  |  |  | *0.5742* |  |  |  |  |  | *0.0357* |
| 0 | 35 | 14 | 6.9 [3.8-NR] | 1 |  |  | 40 | 13 | NR [6.4-NR] | 1 |  |  |
| 1 | 45 | 17 | 6.5 [3.4-NR] | 1.09 | [0.53-2.21] |  | 53 | 17 | NR [6.0-NR] | 1.62 | [0.81-3.25] |  |
| 2 | 35 | 18 | 3.7 [3.2-NR] | 1.48 | [0.73-2.98] |  | 42 | 17 | 6.43 [3.6-NR] | 1.82 | [0.91-3.65] |  |
| 3-4 | 4 | 1 | NR [3.9-NR] | 0.57 | [0.07-4.41] |  | 5 | 2 | 3.96 [2.9-NR] | 3.87 | [.-.] |  |
|  |  |  |  |  |  | **Future perspective** |  |  |  |  |  |  |
|  | *original scores* |  |  |  |  |  |  | *then-test2& then-test3* | |  |  |  |
| **Comorbidity** |  |  |  |  |  |  |  |  |  |  |  |  |
| yes | 192 | 64 | 7.8 [7.1-NR] | 1 |  |  | 187 | 60 | NR [6.4-NR] | 1 |  |  |
| no | 118 | 24 | 7.8 [7.2-NR] | 0.53 | [0.33-0.86] | *0.01* | 115 | 46 | 7.53 [7.2-NR] | 0.68 | [0.45-1.01] | *0.06* |

Table S2: (continued)

|  | **n** | **Event** | **median(95%CI)** | **HR** | **(95%°CI)** | ***p*** | **n** | **Event** | **median(95%CI)** | **HR** | **(95%°CI)** | ***p*** |
| --- | --- | --- | --- | --- | --- | --- | --- | --- | --- | --- | --- | --- |
|  |  | **Without taking recalibration component of RS into account** | | | |  |  | **With recalibration component of RS taken into account** | | | |  |
|  |  |  |  |  |  | **Systemic therapy side effects** |  |  |  |  |  |  |
|  | *original scores* |  |  |  |  |  |  | *then-test1& then-test3* | |  |  |  |
| **Chemotherapy** |  |  |  |  |  |  |  |  |  |  |  |  |
| yes | 137 | 105 | 3.23 [3.13-3.43] | 1 |  |  | 133 | 101 | 3.6 [3.2-6.1] | 1 |  |  |
| no | 182 | 85 | 6.9 [3.83-NR] | 0.48 | [0.36-0.64] | *<0.0001* | 173 | 75 | 6.63 [6.3-NR] | 0.51 | [0.38-0.69] | *<0.0001* |
|  |  |  |  |  |  |  |  |  |  |  |  |  |
|  |  |  |  |  |  | **Breast symptoms** |  |  |  |  |  |  |
|  | *original scores* |  |  |  |  |  |  | *then-test3* |  |  |  |  |
| **Lymph node dissection(LND)** |  |  |  |  |  | *0.018* |  |  |  |  |  | *0.0265* |
| Axillary LND | 97 | 70 | 3.2 [0.6-3.5] | 1 |  |  | 96 | 65 | 3.2 [0.5-5.9] | 1 |  |  |
| Sentinel lymph node biopsy | 105 | 88 | 0.6 [0.4-1.8] | 1.41 | 1.03-1.94 |  | 104 | 82 | 0.5 [0.4-1.8] | 1.37 | [0.99-1.90] |  |
| ALND+SLNB | 22 | 21 | 0.2 [0.1-3.0] | 2.1 | 1.2-3.44 |  | 21 | 19 | 0.2 [0.1-3] | 2.2 | [1.31-3.68] |  |
| No LND | 54 | 42 | 2.7 [0.2-3.3] | 1.33 | 0.90-1.95 |  | 54 | 38 | 0.7 [0.2-3.3] | 1.22 | [0.82-1.83] |  |
| **Professional status** |  |  |  |  |  |  |  |  |  |  |  |  |
| working | 143 | 123 | 0.5 [0.3-0.8] | 1 |  |  | 141 | 110 | 0.5 [0.3-0.6] | 1 |  |  |
| non-working | 130 | 96 | 3.0 [0.7-3.3] | 0.7 | [0.54-0.92] | *0.011* | 128 | 91 | 3.0 [0.7-3.5] | 0.79 | [0.59-1.04] | *0.099* |
| **Quality of life expectations** |  |  |  |  |  | *0.4131* |  |  |  |  |  | *0.0504* |
| improvement | 105 | 82 | 3.0 [0.7-3.3] | 1 |  |  | 103 | 66 | 3.2 [0.6-3.5] | 1 |  |  |
| detrioration | 65 | 50 | 0.5 [0.3-3.3] | 1.03 | [0.72-1.46] |  | 65 | 50 | 0.5 [0.3-3.5] | 1.19 | [0.82-1.72] |  |
| no change | 110 | 90 | 0.4 [0.3-1.8] | 1.21 | [0.89-1.63] |  | 108 | 88 | 0.4 [0.3-1.3] | 1.48 | [1.07-2.04] |  |

Table S2: (continued)

|  | **n** | **Event** | **median(95%CI)** | **HR** | **(95%°CI)** | ***p*** | **n** | **Event** | **median(95%CI)** | **HR** | **(95%°CI)** | ***p*** |
| --- | --- | --- | --- | --- | --- | --- | --- | --- | --- | --- | --- | --- |
|  |  | **Without taking recalibration of RS into account** | | | |  |  | **With recalibration of RS taken into account** | | | |  |
|  |  |  |  |  |  | **Arm symptoms** |  |  |  |  |  |  |
|  | *original scores* |  |  |  |  |  |  | *then-test3* |  |  |  |  |
| **Lymph node dissection(LND)** |  |  |  |  |  | *0.0016* |  |  |  |  |  | *0.0029* |
| Axillary LND | 109 | 87 | 0.5 [0.3-3.1] | 1 |  |  | 109 | 85 | 0.5 [0.3-3.06] | 1 |  |  |
| Sentinel lymph node biopsy | 110 | 66 | 3.5 [0.8-6.2] | 0.59 | [0.43-0.82] |  | 110 | 67 | 3.5 [0.8-6.2] | 0.62 | [0.45-0.86] |  |
| ALND+SLNB | 25 | 21 | 3.1 [0.2-5.9] | 0.92 | [0.57-1.49] |  | 24 | 19 | 3.13 [0.2-5.9] | 0.92 | [0.55-1.51] |  |
| No LND | 62 | 32 | 6.1 [0.5-NR] | 0.52 | [0.34-0.78] |  | 64 | 33 | 6.3 [0.5-NR] | 0.52 | [0.34-0.78] |  |
| **Surgery type** |  |  |  |  |  |  |  |  |  |  |  |  |
| no mastectmoy | 187 | 114 | 3.5 [3.1-6.1] | 1 |  |  | 188 | 112 | 3.9 [3.1-6.1] | 1 |  |  |
| mastectomy | 109 | 86 | 0.5 [0.4-1.5] | 1.51 | [1.14-2.00] | *0.004* | 109 | 86 | 0.5 [0.4-1.5] | 1.52 | [1.15-2.02] | *0.003* |
| **Chemotherapy** |  |  |  |  |  |  |  |  |  |  |  |  |
| yes | 127 | 114 | 0.5 [0.3-1.0] | 1 |  |  | 126 | 100 | 0.5 [0.3-0.7] | 1 |  |  |
| no | 177 | 86 | 3.9 [3.1-6.3] | 0.54 | [0.41-0.71] | *<0.0001* | 179 | 103 | 4.4 [3.0-6.4] | 0.54 | [0.41-0.71] | *<0.0001* |
| **Stage(AJCC)** |  |  |  |  |  | *0.0124* |  |  |  |  |  | *0.0144* |
| 0 | 68 | 36 | 6.1 [0.5-NR] | 1 |  |  | 70 | 37 | 6.1 [2.8-NR] | 1 |  |  |
| 1 | 106 | 65 | 3.4 [0.8-6.3] | 1.12 | [0.74-1.68] |  | 106 | 67 | 3.5 [0.7-6.3] | 1.13 | [0.76-1.70] |  |
| 2 | 94 | 72 | 1.0 [0.3-3.2] | 1.67 | [1.12-2.51] |  | 93 | 67 | 0.7 [0.3-3.3] | 1.63 | [1.09-2.45] |  |
| 3-4 | 13 | 12 | 0.5 [0.2-3.4] | 2.14 | [1.10-4.12] |  | 13 | 12 | 0.5 [0.2-2.8] | 2.34 | [1.21-4.53] |  |
| **Quality of life expectations** |  |  |  |  |  | *0.2246* |  |  |  |  |  | *0.0891* |
| improvement | 116 | 81 | 1.9 [0.5-3.4] | 1 |  |  | 116 | 77 | 1.5 [0.5-3.3] | 1 |  |  |
| detrioration | 68 | 48 | 0.5 [0.3-3.2] | 1.05 | [0.73-1.50] |  | 69 | 51 | 0.5 [0.3-3.5] | 1.12 | [0.78-1.59] |  |
| no change | 122 | 78 | 3.5 [1.0-6.1] | 0.79 | [0.58-1.09] |  | 122 | 77 | 3.9 [0.9-6.4] | 0.77 | [0.56-1.05] |  |
